# Investigation of the Sensing Properties of Lanthanoid Metal–Organic Frameworks (Ln-MOFs) with Terephthalic Acid

**DOI:** 10.3390/molecules29153713

**Published:** 2024-08-05

**Authors:** Denitsa Elenkova, Yana Dimitrova, Martin Tsvetkov, Bernd Morgenstern, Maria Milanova, Dimitar Todorovsky, Joana Zaharieva

**Affiliations:** 1 Faculty of Chemistry and Pharmacy, Sofia University, 1164 Sofia, Bulgaria; yanadim123@gmail.com (Y.D.); nhmt@chem.uni-sofia.bg (M.T.); nhmm@chem.uni-sofia.bg (M.M.); dtodorovsky@yahoo.com (D.T.); 2 Inorganic Solid State Chemistry, Saarland University, Campus Geb. C4 1, 66123 Saarbrücken, Germany; bernd.morgenstern@uni-saarland.de

**Keywords:** lanthanoids, europium, terbium, metal–organic frameworks (MOFs), sensors

## Abstract

The solvothermal synthesis of LnCl_3_^.^nH_2_O with terephthalic acid (benzene-1,4-dicarboxylic acid, H_2_BDC) produced metal–organic frameworks (LnBDC), [Ln_2_(BDC)_3_(H_2_O)_4_]_∞_, where Ln = Sm, Eu, Tb, and Dy. The materials obtained were characterized by a number of physico-chemical techniques. The influence of the ionic radius of the lanthanides on the microstructural characteristics of the Ln-MOFs was evaluated by performing Rietveld refinement. The MOFs obtained were tested as fluorescent sensors for numerous cations and anions in water. The highly luminescent EuBDC and TbBDC demonstrated multi-responsive luminescence sensing functions to detect Ag(I), Fe(III), Cr(III), and Cr(VI), which are essential for their environmental applications. By applying the non-linear Stern–Volmer equation, the fluorescent quenching mechanism was determined. The stability of the obtained materials in water in a wide pH range (acidity pH = 4 and alkalinity pH = 9 solutions) was confirmed.

## 1. Introduction

Metal–organic frameworks (MOFs), also known as potential porous coordination polymers, are an emerging class of molecular materials. Due to their structural and functional tenability, investigations based on MOFs are among the fastest-growing scientific fields. These crystalline hybrid materials are built by means of coordination bonds between inorganic metal-containing units and organic ligands. In their construction, secondary building units (the metal-containing nodes) and binding ligands (the organic linkers) can be distinguished [1]. This allows modification and control of the pore size, as well as their functionality. The structure obtained can be a one-, two-, or three-dimensional coordination network with potential voids [2].

The lanthanoids Sm, Eu, Tb, and Dy and their trivalent ions possess fluorescence properties in the visible region due to Stokes shift upon activation by near-UV light. Since f→f transitions according to Laporte’s selection rules are forbidden, their direct activation is impractical due to the low molar absorption coefficients leading to negligible intrinsic fluorescence [3]. This deficiency can be overcome by incorporating the ions into organic and inorganic materials, such as MOFs, which possess groups/ions capable of carrying out charge transfer to the photoactive metal (antenna effect).

Terephthalic acid (benzene-1,4-dicarboxylic acid, H_2_BDC) has been studied as a ligand leading to luminescence enhancement of Ln(III) ions [4]. More than 20 different crystal structures of Ln(III) ions synthesized and characterized with terephthalic acid are an illustration of the diversity obtained with a single ligand by varying the synthesis conditions. Both mixed-ligand [5,6] and mixed-metal MOFs [7,8] have been prepared with terephthalic acid.

Among the most common structures obtained with terephthalic acid and Ln(III) is the one presented and discussed in this study, corresponding to the empirical formula [Ln_2_(BDC)_3_(H_2_O)_4_]_∞_, triclinic, P−1. First, Reineke and co-workers [9] published this structure with Tb(III) and studied its fluorescence properties. Later, Haquin and co-workers studied its NIR luminescence with Er(III) in 2009 [10], and in 2013, their work included five more Ln(III) ions (Tb, Dy, Sm, Eu, and Gd), with a focus on the luminescence color of those materials for potential LED applications [11]. In 2009, Daiguebonne et al. reported the synthesis and characterization of coordination polymers with the general formula [Ln_2_(BDC)_3_(H_2_O)_4_]_n_, with Ln = Y, La-Lu (except Pm) [12], and expanded the study in 2009 [13] with mixed-metal structures and investigation of their tunable emission. Their polymers also correspond to the structure investigated in this work. Two more reports on the structure in question, one focused on the Ce analogue [14] and the other focused on structural studies involving a comparison with several different structures where the number of coordinated water molecules was not four [15], have been published. In 2013, Cadiau and co-workers suggested materials based on this structure for a radiometric nanothermometer [16]. Interestingly, these materials have also been suggested to be used for gunshot residue (GSR) luminescent marking [17]. A mechanochemical approach was also reported as a method for their synthesis in 2020 [18].

Investigations on two potential applications of the Ln-MOF mentioned before are scarce in the literature: (i) as catalysts or catalyst carriers in advanced oxidation processes for water and air purification (to be mentioned, our group recently reported some promising results in this field [19]) and (ii) as sensor materials for detecting water pollutants.

Although MOFs are attracting interest as sensor materials [20,21,22,23,24,25,26,27], as far as we are aware, only Zhang and co-workers investigated MOFs with the composition mentioned for detecting picric acid (TNP) [28]; recently, Donghan et al. discussed the fluorescence turn off–turn on continuous response of dual-lanthanide metal–organic frameworks for selectively detecting fluoroquinolone antibiotics [29]. So, the focus of this work is on the investigation, in detail, of Ln-MOFs with the composition [Ln_2_(BDC)_3_(H_2_O)_4_]_∞_ (Ln = Sm, Eu, Tb, Dy) as potential sensors for water pollutants. A detailed investigation of the stability of the materials in water, as well as in environments with different acidity levels, and of their activity in different solvents and in the presence of different ions (cations and anions) is included. The latter is rarely discussed in the literature but is important for sensor materials. Structural characteristics play an important role in this type of application; especially, the crystallite size and microstrains play a significant role in the intensity of luminescence, which, in its turn, is essential for sensor application. So, even for isostructural MOFs, these characteristics are essential for sensor performance in a water suspension, and this is the reason information about all samples is extracted by applying Rietveld refinement, which brings novelty to the work, along with all the investigation related to sensor properties. An important aspect of this investigation is the detailed description of non-linear Stern–Volmer data and the possible mechanisms of quenching.

## 2. Results and Discussion

### 2.1. Crystal Structure of DyBDC–Single Crystal (SC)

The experimental details of single-crystal diffraction measurements, as well as corresponding CCDC deposition numbers, are summarized in Table S1 [30]. The data can be obtained free of charge from http://www.ccdc.cam.ac.uk/conts/retrieving.html (or from the Cambridge Crystallographic Data Centre, 12, Union Road, Cambridge CB2 1EZ, UK; fax: +44-1223-336033).

**Figure 1:**
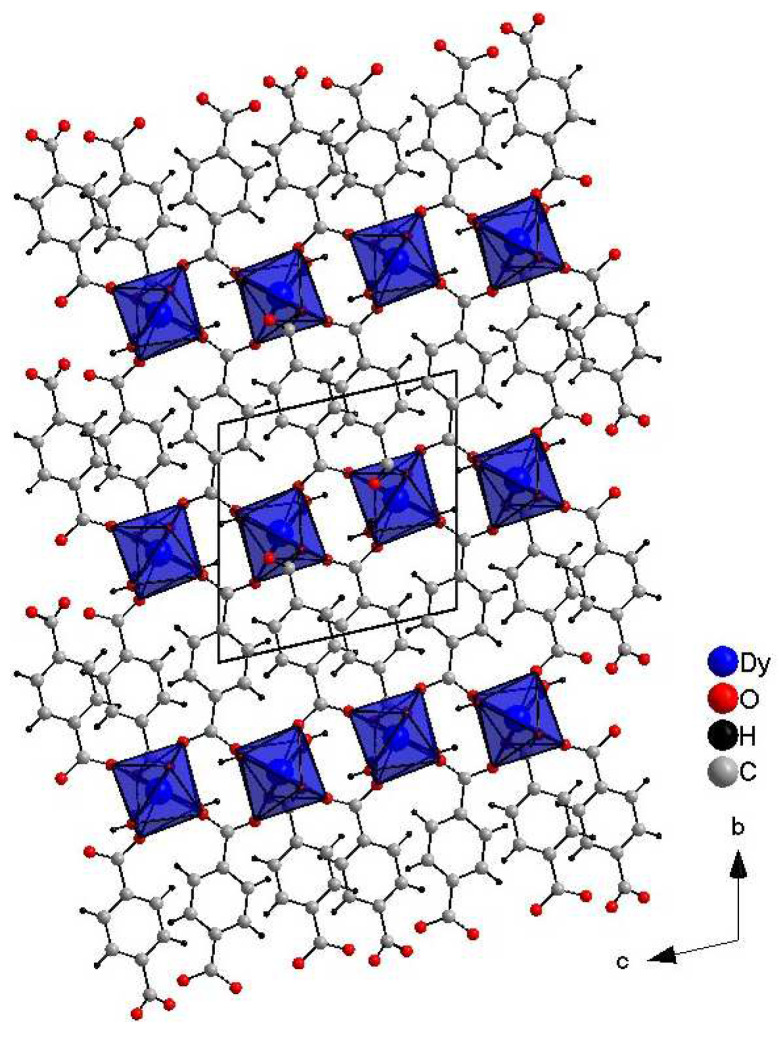
Crystals structure images of DyBDC-SC.

Crystals suitable for single-crystal XRD measurements were obtained The crystal packing viewed along the a-axis of DyBDC (DyBDC-SC) is shown in Figure 1. The obtained material was isostructural to the Nd [15], Tb [9], and Er [15] analogues reported earlier and crystallizes in the triclinic P−1 space group. To the best of our knowledge, this is the first report of a Dy analogue structure obtained by single-crystal diffraction. The Dy(III) ions were the center of a distorted DyO_8_ (distortion index 0.04243), where the six oxygen atoms belonged to the adjacent terephthalic acid ligand and the other two to the water molecules. The effective coordination number was found to be 7.37, which is due to the fact that the Dy–O distance involving the oxygen atoms of the water molecules is slightly longer, in the range of 2.491(4) Å—2.513(4) Å (average 2.502(4) Å), while the bonds involving the oxygen atoms from the terephthalic acid are in the range of 2.268(3) Å—2.481(4) Å (average 2.340(4) Å).

### 2.2. Powder X-ray Diffraction

**Figure 2:**
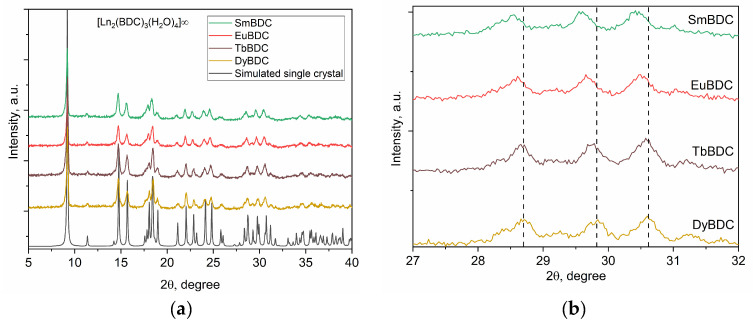
(**a**) XRD patterns of the as-prepared LnBDC MOFs compared to the simulated XRD pattern of the DyBDC-SC and (**b**) a close look at the 27—32 2θ part confirming the change in the unit cell parameters.

The powder XRD patterns of all obtained MOFs are presented in Figure 2, which confirms that all the materials were isostructural to each other and to the DyBDC-SC. The phase analysis confirmed that all the XRD peaks belonged to the [Ln_2_(BDC)_3_(H_2_O)_4_]_∞_ phase, with no crystalline impurity phases.

Insight into the crystal structure and microstructural characteristics of the obtained powders was gained by performing full-profile Rietveld refinement. The results are summarized in Table S2, while the Rietveld refinement profiles are presented in Figure S1. The unit cell volume and unit cell parameters (Figure 2b) of the MOFs slowly decreased from the Sm to the Dy analogue, which is in good agreement with the change in the ionic radius due to lanthanide contraction. Significant differences were observed in the microstructural characteristics of the samples. Despite being prepared by applying the same procedure, a significant difference in the crystallite sizes of the obtained MOFs, with a clear trend of size reduction from Sm to Dy, was observed. In spite of the latter trend mentioned, the microstrain values were virtually the same within the standard deviation.

### 2.3. Morphology

**Figure 3:**
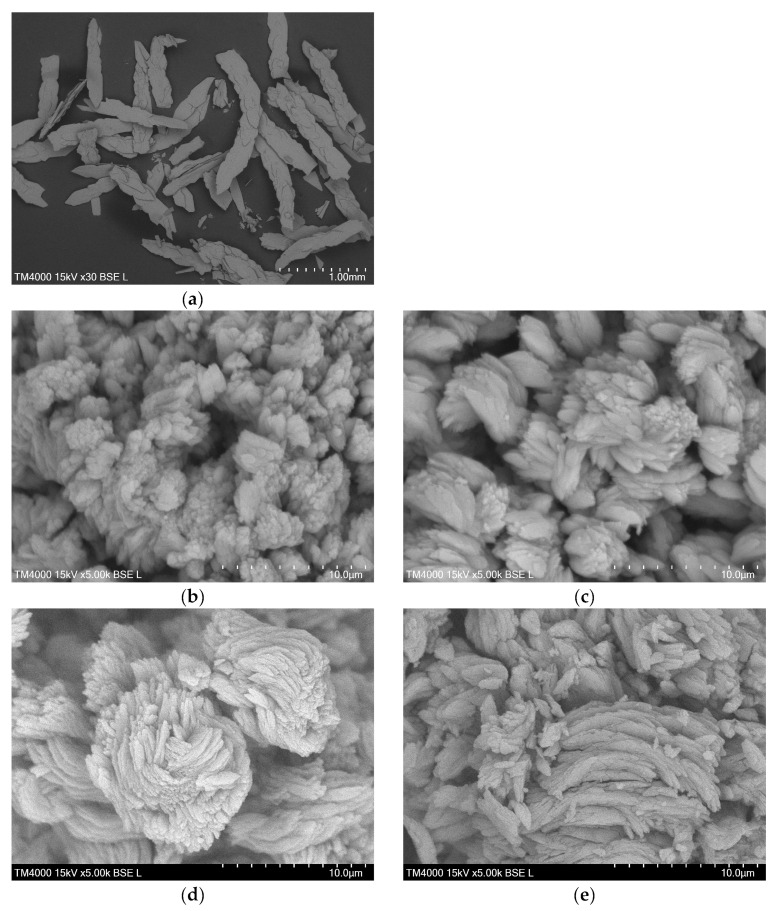
SEM images of (**a**) single crystals of DyBDC-SC and polycrystal samples of (**b**) SmBDC, (**c**) EuBDC, (**d**) TbBDC, and (**e**) DyBDC.

The SEM image of the DyBDC-SC (Figure 3a) revealed flat and lamellar crystals with a sheet-like shape and not-so-clearly-defined crystal edges. The SEM images of the polycrystalline samples (Figure 3b–e) showed similar morphological features agglomerated into flower-like pattern lamellae. The particles were polydispersed in size. Despite that, it was noticeable that the smallest average lamellae size was that of the EuBDC, followed by that of the SmBDC, while the TbBDC and the DyBDC were almost identical in size.

### 2.4. Optical Properties

**Figure 4:**
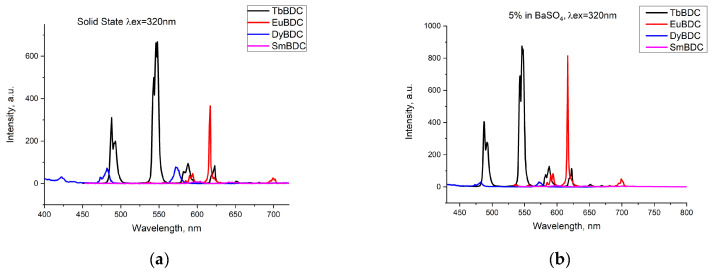
Comparison of the emissions registered from different types of LnBDC: (**a**) pure samples in a solid state; (**b**) 5 wt% LnBDC in BaSO_4_.

Emissions of the powdered samples with four different Ln(III) ions were recorded (Figure 4a) under the same conditions (λex = 320 nm; ex/em slit 5/1.5 nm; data interval 1.0 nm; average time 0.10 s; PMT voltage 800 V). The most intensive emission was detected for TbBDC, followed by EuBDC. Emission from SmBDC and DyBDC was also present, as can be seen from the normalized spectra (Figure S2), but obviously, terephthalic acid is not a good sensitizer of their luminescence, observed as well in [11]. A possible explanation is the small energy gap possessed by Dy(III) and Sm(III) [31], which favors nonradiative deactivation, especially in the presence of coordinated water molecules. The energy of the lowest triplet state of terephthalic acid, 23,530 cm^−1^ [11], happens to be suitable for the energy gap between the emitting level ^5^D_4_ of Tb(III) [32]; therefore, good luminescence was detected for TbBDC. As a difference, a lower emission by EuBDC in comparison to that by TbBDC was noticed. To ensure the lack/existence of self-quenching effects, the luminescence of the samples in a mixture with BaSO_4_ was recorded (LnBDC in BaSO_4_ 5 wt%). It was clearly observed (Figure 4b) that the emissions from TbBDC and EuBDC now were of almost equal intensity. This observation is evidence of the self-quenching effect, significantly influencing the EuBDC emission. Following the results observed, subsequent measurements and experiments on sensor properties were focused mainly on Tb(III) and Eu(III) samples.

Lifetime measurements in the solid state and in a water suspension also demonstrated longer lifetimes for TbBDC, both in the solid state and in the water suspension (Table 1). The decay curves were fitted monoexponentially. The values obtained for TbBDC and EuBDC are in agreement with the literature data for this composition [11]. No significant difference was observed when the lifetimes were measured in mixtures with BaSO_4_.

### 2.5. Sensor Properties

As mentioned before, the experiments for sensor properties were performed only with EuBDC and TbBDC samples because of their more intense luminescence. As the specific surface area and the pore size are important parameters for the sensor performance of materials, N_2_ adsorption–desorption analysis of TbBDC and EuBDC was performed. The obtained isotherms (Figure S3) were of type IV, with an H3-type hysteresis loop according to the IUPAC classification [33]. This indicated weak adsorbent–adsorbate interactions, and it is typical for nonporous or microporous solids, which is in good agreement with the literature data for these MOFs. The calculated specific surface area, the average pore volume, and the total pore volume are summarized in Table S3. The calculated specific area was 14.60 and 14.75 m^2^/g for TbBDC and EuBDC, respectively. The values obtained are significantly higher than the literature data for the same structure [34], confirming that the synthesis conditions and resulting morphology play a significant role in the sensor’s properties and performance ability.

Preliminary experiments in order to determine the best concentration of the samples in water suspensions were performed (Figure S4). As a result, the concentration of 0.5 mg/mL for the samples was selected for further research. This concentration ensures suspension stability and helps to avoid self-quenching of Ln(III) ions.

#### 2.5.1. Stability of the Water Suspensions of the Samples

**Figure 5:**
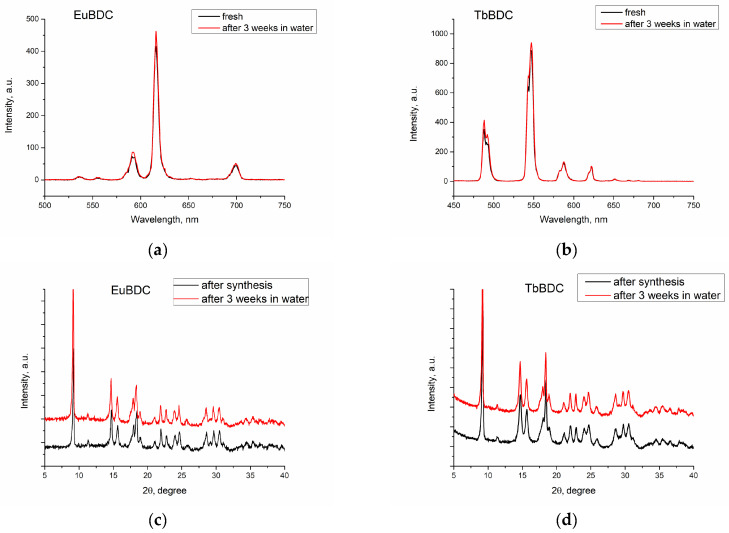
Luminescence spectra of freshly prepared and 3-week-aged suspensions of (**a**) EuBDC and (**b**) TbBDC. Powder XRD just after synthesis and after 3 weeks’ stay in water for (**c**) EuBDC and (**d**) TbBDC.

Luminescence was recorded, both of freshly prepared and of 3-week-aged suspensions (Figure 5a,b). First, the suspension was centrifugated. Next, the sample was dried. Finally, the phase homogeneity was determined by powder XRD (Figure 5c,d). With this procedure, the stability in water and after multiple ultrasound treatments was evaluated.

The results confirmed that both EuBDC and TbBDC samples are stable in water for at least 3 weeks. Luminescence was kept stable, and powder X-ray diffraction showed no change in the sample phase composition.

#### 2.5.2. Behavior in Different Solvents

**Figure 6:**
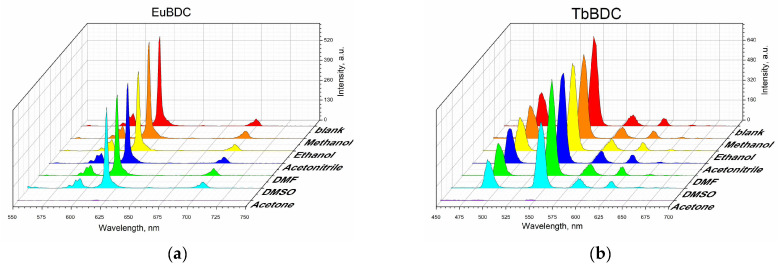
Luminescence measurements under the same conditions of 0.5 mg/mL of suspension in different solvents: (**a**) EuBDC and (**b**) TbBDC.

Suspensions in different solvents were prepared, and their luminescence was measured according to the procedure described in Section 3 (Materials and Methods). Both samples were sensitive to acetone, and the luminescence was fully quenched in that solvent (Figure 6), but the luminescence recovered after the removal of acetone (Figure S5). Although some studies have focused on sensing acetone in water [35,36,37,38], it is not considered a water pollutant, due to its volatility.

#### 2.5.3. Behavior in Media with Different Acidity Levels

An important property of potential sensors is their behavior in media with different acidity levels. The pH value of natural waters depends on many variables, but usually, waste waters from mines have a pH of about 2 to 3; swamps, 4 to 6; groundwaters, 5 to 7; rivers, 7–8; fresh lakes, 7–9.5; and oceans, 8–8.5 [39]. So, obviously, it is crucial to know the sensor behavior in these pH ranges.

**Figure 7:**
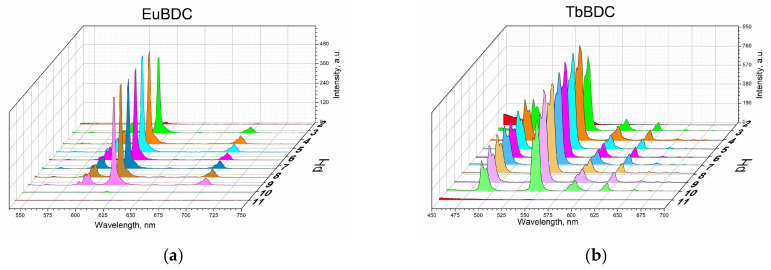
Luminescence measurements under the same conditions of 0.5 mg/mL of suspension at different pH values: (**a**) EuBDC and (**b**) TbBDC.

Measurements were performed according to the procedure described in Section 3 (Materials and Methods). From Figure 7, it is seen that both EuBDC and TbBDC behave similarly and have stable luminescence in the pH range of 4 to 9. In both cases, it was observed that there was a decrease in the luminescence at a pH of about 3 and about 10 and almost full loss of luminescence at a pH below 3 and over 10. Such a drastic loss of luminescence is most likely due to the destruction of the MOF. This was additionally confirmed with PXRD of the TbBDC sample at pH 1.5 and pH 11.90 (Figure S6). At low pH, reflections from H_2_BDC were observed, which is a clear indication of the destruction of the original MOF. At high pH, no crystal phase could be observed, suggesting a destruction process and the presence of the amorphous hydroxide of Ln(III). Nevertheless, both samples demonstrated a good working range for drinking waters, and it is worth continuing the tests.

#### 2.5.4. Behavior in the Presence of Different Cations and Anions

Emissions from the samples were measured under the same conditions, and to detect sensor properties, the maximum emission wavelength was applied, at 616 and 547 nm for EuBDC and TbBDC, respectively. The procedure is described in Section 3 (Methods and Materials).

**Figure 8:**
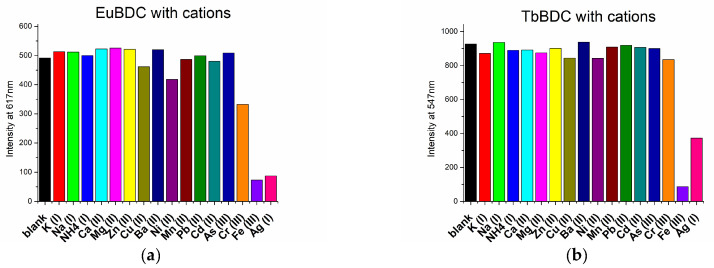
Maximum emission observed at 617 nm for EuBDC (**a**) and at 547 nm for TbBDC (**b**) in the presence of different cations (concentration 1 × 10^−3^ mol/L, nitrate salts, λ_ex_ = 320 nm).

**Figure 9:**
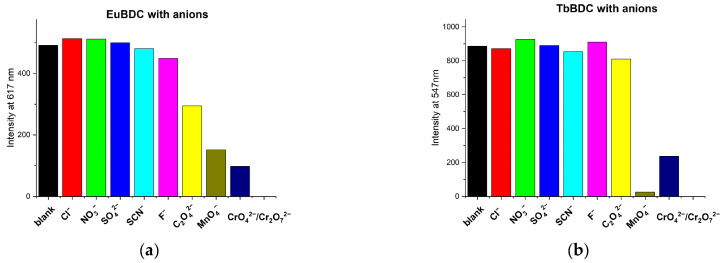
Maximum emission at 617 nm for EuBDC (**a**) and at 547 nm for TbBDC (**b**) in the presence of different anions (concentration 1 × 10^−3^ mol/L, sodium and potassium salts, λ_ex_ = 320 nm.

The sensor properties of both EuBDC and TbBDC were tested for 16 different cations (Figure 8). Based on the emission registered, the samples showed the highest sensitivity to Fe(III), followed by Ag(I). Sensitivity to Cr(III) differed essentially, and EuBDC can be used as a sensor for Cr(III). The results with anions were promising, too (Figure 9); namely, (i) both samples were sensitive to MnO_4_^−^ and CrO_4_^2−^/Cr_2_O_7_^2−^, (ii) EuBDC demonstrated a decrease in luminescence in the presence of C_2_O_4_^2−^ anions, and (iii) TbBDC happened to be more sensitive to MnO_4_^−^.

The pH levels of the solutions of the ions were measured in order to exclude the influence of the acidity of the environment (Table S4). As data on the potential health effects of manganese remain uncertain, particularly relating to the form of manganese that may be of concern, and it was difficult to determine a suitable health-based value at this time [40], we did not perform experiments with it. As long as the oxalate ions form low-solubility products with cations usually present in water, they are not considered a threat. The following sections present research results with the ions of Ag(I), Cr(III), Fe(III), and Cr(VI).

#### 2.5.5. Silver(I) Sensitivity of EuBDC and of TbBDC

Average silver concentrations in natural waters according to the World Health Organization are 0.2–0.3 μg/L [41]. Water treated with silver may have ion levels of 50 μg/L or higher. Higher levels of silver ions, up to 0.1 mg/L (a concentration that gives a total dose over 70 years of half the human no-observed-adverse-effect level (NOAEL) of 10 g), could then be tolerated without risk to health [41]. So, silver is an element of interest, and a concentration above 9.27×10^−7^ mol/L is considered dangerous for humans, and further tests will be performed. The procedure is explained in Section 3.

**Figure 10:**
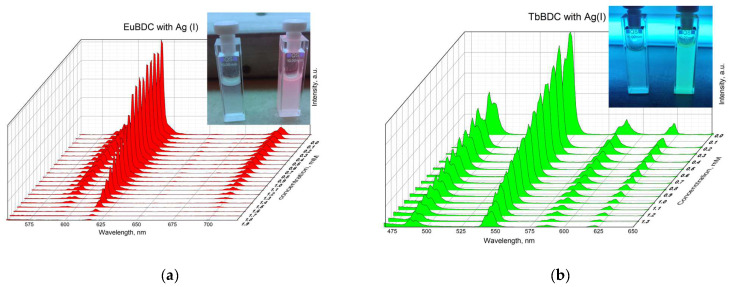
Luminescence of (**a**) EuBDC (inset: picture of the suspension under a UV lamp with max. 254 nm with and without a quencher) and (**b**) TbBDC at different concentrations of Ag(I) (inset: picture of the suspension under a UV lamp with max. 254 nm with and without a quencher).

**Figure 11:**
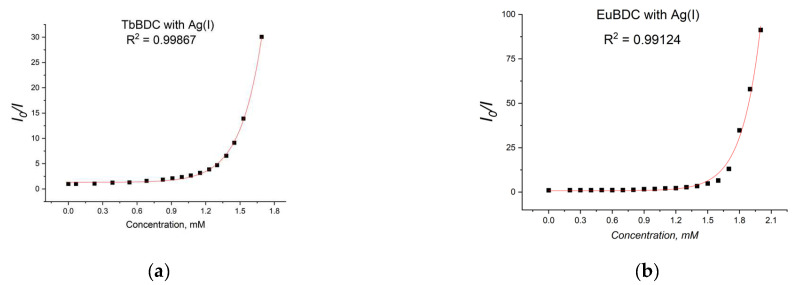
Stern–Volmer curves for (**a**) EuBDC and (**b**) TbBDC (λex = 320 nm). Intensity at 547 nm in the absence of a quencher, divided by the intensity in the presence of a quencher; baseline correction applied.

A gradual decrease in luminescence was observed with an increase in the concentration of Ag(I) (Figure 10). By plotting the intensity in the absence of a quencher versus the intensity in the presence of a quencher (I_0_/I) to the concentration of Ag(I) ions in both MOFs, exponential curves were obtained (Figure 11). In Section 3.5.4 are described all the equations used for the discussion of the following results. Equation (7) was used to describe the data from Figure 11.

From Figure 11a, for EuBDC with Ag(I), exponential fitting of I_0_/I = 0.00123e^5.61691[Q]^ + 0.8033 was obtained. The Stern–Volmer constant K_sv_ was calculated to be 6.908 M^−1^ (R^2^ = 0.99124). For TbBDC (Figure 11b), I_0_/I = 0.0037e^5.30066[Q]^ + 1.30927, and K_sv_ was calculated to be 19.61 M^−1^ (R^2^ = 0.99867). Both values for K_sv_ were low, but one has to keep in mind that there are no data specifically for the K_sv_ for Ag(I) when MOF materials are used as sensors.

**Figure 12:**
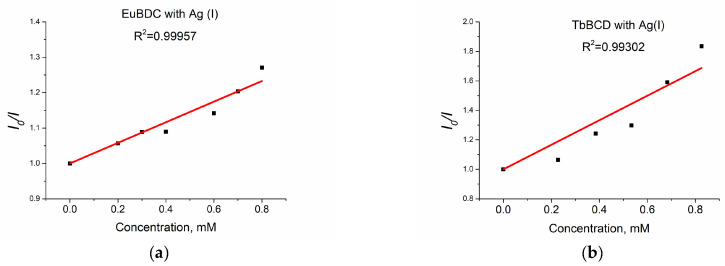
(**a**) EuBDC and (**b**) TbBDC linear dependency interval.

When solutions with concentrations up to 0.8 mM of Ag(I) were tested, a linear dependence with much higher values of K_sv_ (Figure 12a) was observed.

The values obtained for EuBDC were K_sv_ = 2911 M^−1^ and R^2^ = 0.99957 and for TbBDC were K_sv_ = 8322 M^−1^ and R^2^ = 0.99302. These values were much higher compared to those calculated from the exponential fit, suggesting that both materials can be used as sensors only at low concentrations. It is clear that TbBDC performs much better than EuBDC, with a higher value for Ksv and a lower value for LOD (88 µM for TbBDC compared to 104 µM for EuBDC) (Table S5). The LOD is 3σ/slope, where σ is the standard deviation of the blank sample measured 10 times, and the slope is taken from the intensity-versus-concentration plot in the linear region.

**Figure 13:**
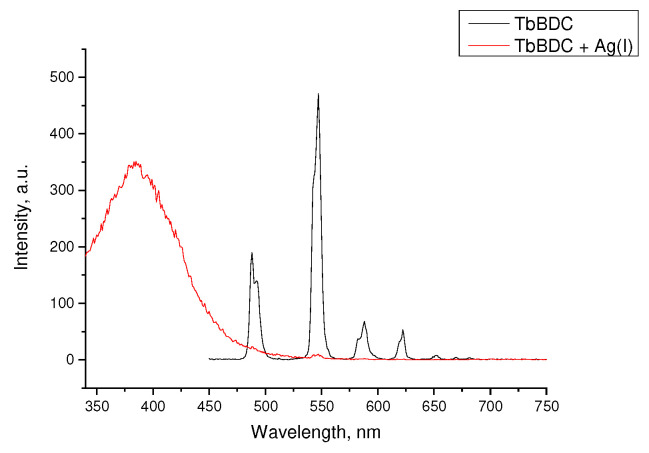
Luminescence spectra of TbBDC in the absence of Ag(I) and at concentrations over 1.8 mM.

When the concentration of silver ions was high enough to quench almost 100% of the luminescence, emission from the linker was clearly observed (Figure 13). All attempts to recover the luminescence after washing the sample were unsuccessful. This may suggest a more complex character of the quenching and explain the high exponential dependence at higher concentrations and unusual K_sv_ values.

It can be suggested that at low concentrations, dynamic quenching plays a major role, and at a concentration higher than 0.8 mM, static quenching called “sphere of action” is also present. Moreover, as the luminescence did not recover after full quenching, we again explored PXRD, and patterns of the silver MOF [42] with terephthalic acid were observed (Figure S6). Obviously, partial destruction of the original MOF also plays a role and explains the unusual Stern–Volmer dependence.

#### 2.5.6. Chromium (III) Sensitivity of EuBDC

**Figure 14:**
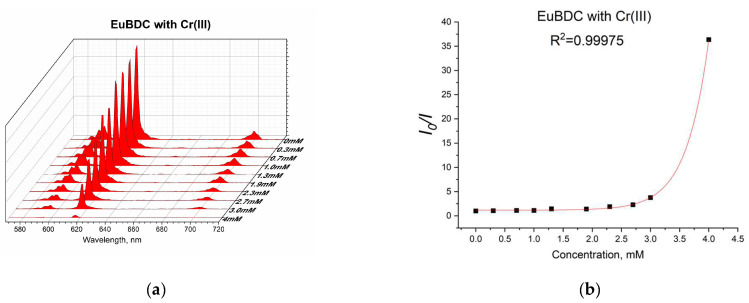
(**a**) Luminescence at different concentrations of Cr(III) and (**b**) exponential fit of the data.

Chromium(III) was tested only with EuBDC (Figure 14a) as EuBDC is more sensitive to it (see Figure 8). A guideline value (GV) of 50 μg/L has been proposed for the total chromium content based on achievability by current treatment technologies, measurability by analytical methods, and toxicology [43]. The risk assessment for chromium in drinking water is based on recent high-quality data from chronic drinking-water carcinogenicity and mode-of-action studies for Cr(III) and Cr(VI). As chromium is usually found in drinking water at an average concentration of 1 μg/L, which is below the GV, in general, the monitoring and inclusion of Cr in drinking-water regulations and standards would only be necessary if there were indications that a problem might exist [43].

**Figure 15:**
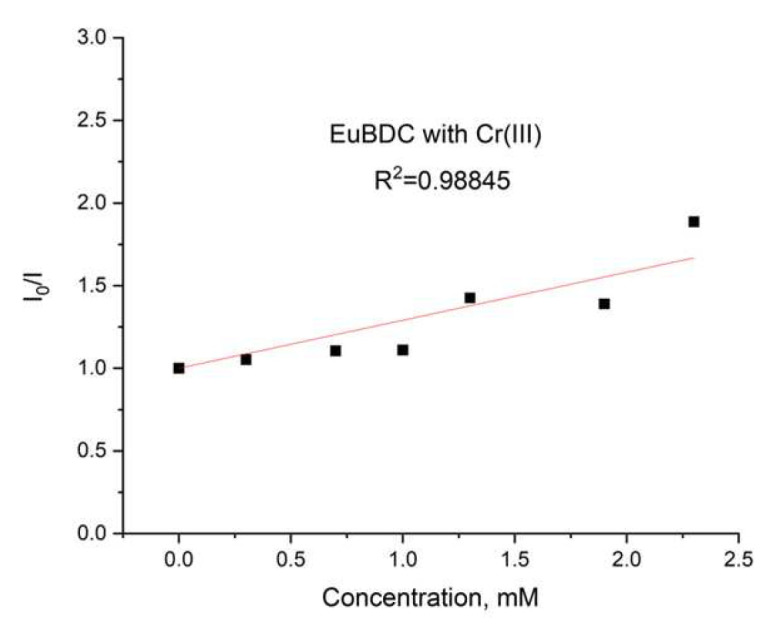
Stern–Volmer curve for EuBDC: a linear dependency interval for Cr(III).

Again, an exponential curve was observed with fitting I_0_/I = 0.00106e^2.60329[Q]^ + 1.17343 (Figure 14b). The value of K_sv_ was calculated to be 2.76 M^−1^ (R^2^ = 0.99975). This is similar behavior to the one observed in the case of Ag(I). Linear dependency at lower concentrations of up to 2.4 mM (Figure 15) and a Ksv value of 3440 M^−1^ was observed, suggesting again that this sensor is appropriate only for low concentrations of Cr(III). In this case, as well as with Ag(I), after quenching, full recovery of luminescence was not possible. Most likely, again, a destruction process was involved, so the application of these materials as sensors for Ag(I) and Cr(III) will be difficult and will limit it in a narrow concentration range. Moreover, the LODs calculated from the linear dependence for both materials and ions were high (see Table S5). However, this unusual form of the exponential curve can be used to recognize the influence of MOF destruction processes. It is also important to know the behavior of a given material toward any ion potentially present in water in order to fully appreciate its capabilities.

#### 2.5.7. Iron (III) Sensitivity of TbBDC

According to the WHO [44], the median iron concentration in rivers has been reported to be 0.7 mg/L. In anaerobic groundwater where iron is in the form of iron(II), concentrations will usually be 0.5–10 mg/L, but concentrations up to 50 mg/L can sometimes be found as well. Concentrations of iron in drinking waters are normally less than 0.3 mg/L but may be higher in countries where various iron salts are used as coagulating agents in water treatment plants and where cast iron, steel, and galvanized iron pipes are used for water distribution. Taste is not usually noticeable at iron concentrations below 0.3 mg/L, although turbidity and color may develop in piped systems at levels above 0.05–0.1 mg/L. Laundry and sanitaryware will get stained at iron concentrations above 0.3 mg/L. Allocation of 10% of the provisional maximum tolerable daily intake of drinking water provides a value of about 2 mg/L, which does not present a hazard to health. The taste and appearance of drinking water will usually be affected below this level, although iron concentrations of 1–3 mg/L can be acceptable for people drinking anaerobic well water [44]. Apparently, this cation is of great interest, as quite a lot of studies have investigated Ln-MOFs as sensors of Fe(III) [21,45,46,47,48,49,50,51,52].

**Figure 16:**
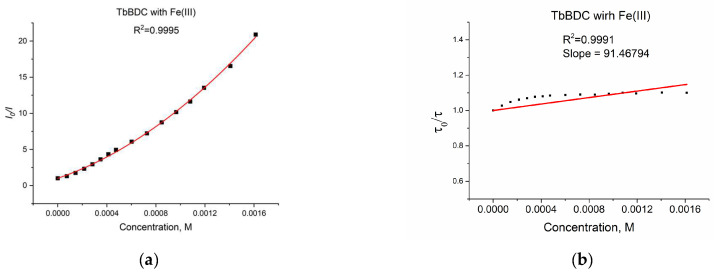
(**a**) Intensity at 547 nm in the absence of a quencher, divided by the intensity in the presence a of quencher (λex =320 nm); baseline correction applied. (**b**) Lifetime value in the absence of a quencher, divided by lifetime values in the presence of a quencher.

A plot of the data from a luminescent sensor experiment with Fe(III) can be seen in Figure 16a.

As discussed in Section 3.5.4, when both static quenching and dynamic quenching were combined, we observed both quenching by collisions and quenching by complex formation with the same quencher. The Stern–Volmer equation is a second-order polynomial curve (Equation (3)). The fitting was good, with R^2^ = 0.9995 (Figure 16a).

**Figure 17:**
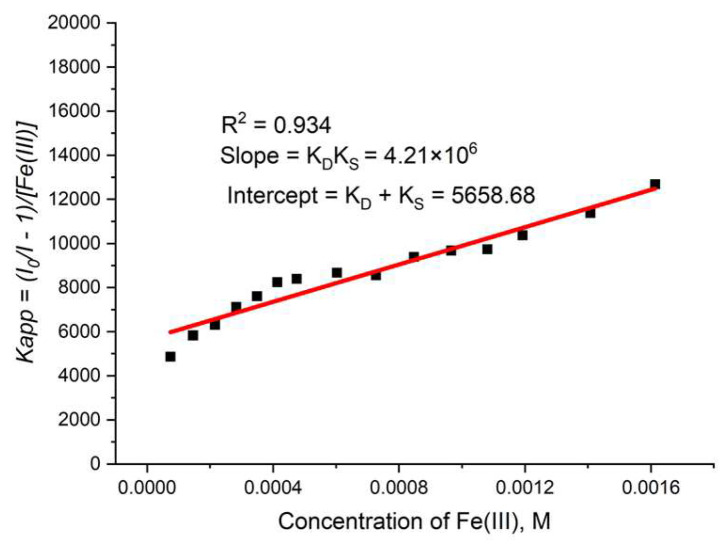
Graphical separation of K_S_ and K_D_ for TbBDC with Fe(III).

During the measurement, the lifetime was also determined (Figure 16b) for each concentration of the quencher. We observed a small value for the slope, suggesting that the dynamic quenching constant is low and, obviously, we have bigger static quenching. We can see from Figure 16b that dynamic quenching played a role again at low concentrations and above 0.4 mM. We can use the modified Equation (3) to allow a graphical separation of K_S_ and K_D_ for the apparent quenching constant (Equations (4) and (5)) shown in Figure 17.

The possible value for K_S_ was 4777 or 881 M^−1^. Lifetime data (Figure 16b) suggested that the higher value is more reasonable for a static quenching constant.

**Figure 18:**
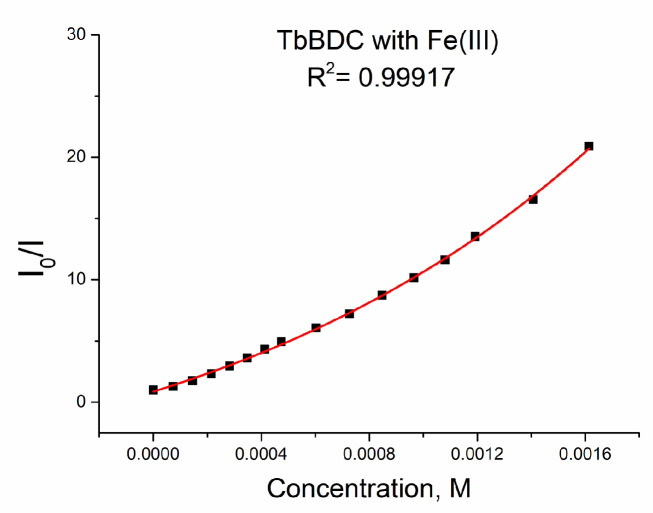
Exponential fit of the data for TbBDC with Fe(III).

The data could be fitted exponentially, too, with a similar value of R^2^ of 0.99917 (Figure 18).

When we used exponential fitting, we could determine K_sv_ (see Equation (5)), so the value was 6903 M^−1^. This value is in good agreement with literature data, where values from 1000 M^−1^ to 13,000 M^−1^ can be found for Fe(III) sensing [21,45,46,47,48,49,50,51,52]. Of course, data can be fitted linearly up to concentrations of 1.0 mM, with reasonable values of R^2^ = 0.9939 and K_sv_ = 8924 M^−1^ (Figure S8). The LOD was 32.5 µM, which is much better than that calculated for Ag(I) and Cr(III). A comparison of the types of data manipulations is summarized in Table S5.

#### 2.5.8. Chromium (VI) sensitivity of TbBDC

As discussed already, chromium is explored in water as the total for both Cr(III) and Cr(VI). Interestingly, only EuBDC demonstrated sensitivity to Cr(III), which can be used as a tool to determine different forms of chromium in water, as for Cr(VI), both EuBDC and TbBDC were sensitive, and tests were performed with the better-luminescent TbBDC.

**Figure 19:**
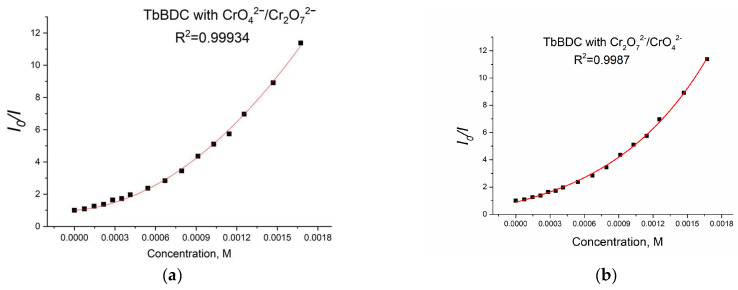
Polynomic (**a**) and exponential (**b**) fit of the data for TbBDC with Cr(VI).

**Figure 20:**
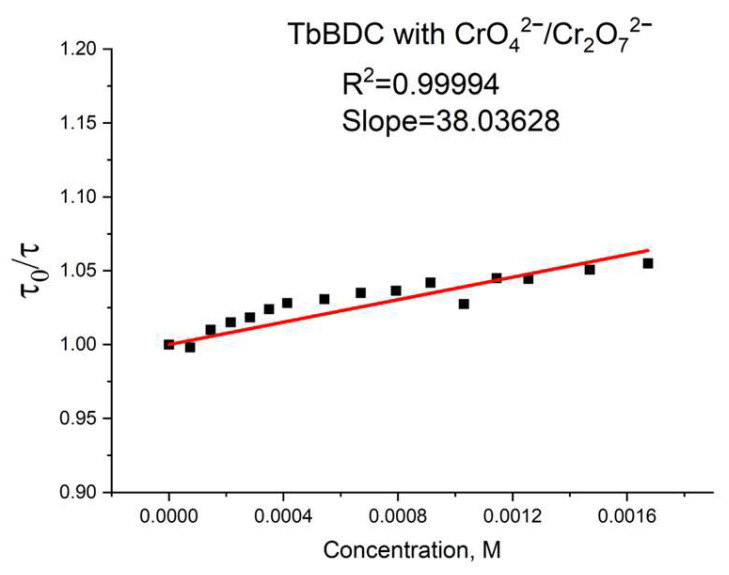
Lifetime value in the absence of a quencher, divided by lifetime values in the presence of a quencher.

Again, polynomic fit and exponential fit can be applied to the data, both with high R^2^ (Figure 19), but in this case, we could not obtain real solutions from the polynomic fit when using Equations (4) and (5) (Figure S9). Apparently, the value from the intercept is mainly from static quenching. Lifetime measurements (Figure 20) suggested independence of the lifetime from the concentration, as the slope was low and we observed dynamic quenching only at low concentrations. Most likely, static quenching plays a major role in both types: (i) the formation of a nonfluorescent ground state confirmed by the independence of the lifetime and (ii) “sphere of action”, confirmed by the exponential fit.

Furthermore, the calculated K_sv_ value from the exponential fit of the data was 2093 M^−1^. This value is also in good agreement with literature data [46,53,54,55]. Linear fit (Figure S10) gives a K_sv_ value of 2749 M^−1^ and an LOD value of 49.5 µM, suggesting there is good potential of this material as a sensor for Cr(VI). It is worth mentioning that the different type of exponential fitting for Cr(III) (see Figure 14b) is one more way to separate the two oxidation forms when tested.

#### 2.5.9. Possible Quenching Mechanisms

A more detailed discussion of the mechanisms of luminescence quenching in the presence of acetone, Ag(I), Cr(III), Fe(III), and CrO_4_^2−^/Cr_2_O_7_^2−^ is presented in this section.

A possible mechanism of quenching with acetone is Förster resonance energy transfer, i.e., a nonradiative transfer of energy from a donor to an acceptor molecule (observed if the acceptor molecule possesses high-energy vibrational states, e.g., it is an organic molecule with functional groups such as –OH, –CN, and –NO_2_) [37]. After the removal of acetone, full recovery of the luminescence is observed (Figure S5). Acetone has an observable absorption in the 225–325 nm region, while the other solvents do not possess an absorption band at 320 nm [56]. Based on this, energy transfer between the ligand and acetone can be suggested, indicating a sort of competitiveness between the compound excitation and the acetone absorption. The latter causes a decrease in the luminescence intensity. As previously mentioned, acetone is not considered a water pollutant, and experiments with different concentrations were not performed. In spite of that, we considered the elucidation of the quenching mechanism, as well as of the luminescence recovering after acetone removal, an important move for further experiments.

Regarding the change in the luminescence of MOFs caused by the metal ions, four different mechanisms have been reported, taking into consideration (i) a weak interaction between the metal ions and the free Lewis basic sites within the organic ligands of the MOF, (ii) an ion exchange between the central metal ion and the targeted ions, (iii) destruction of the framework, and (iv) absorption of excitation light, FRET, and other excited-state processes [57].

**Figure 21:**
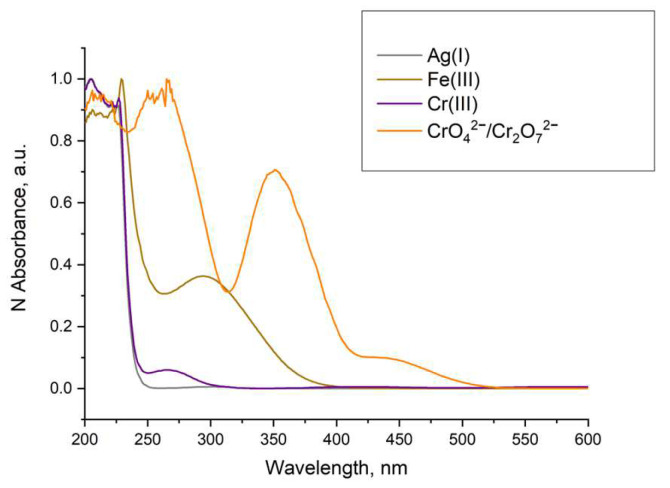
Normalized absorption of solutions with concentration 1×10^−3^ mol/L.

In the case of Ag(I) and Cr(III) quenching, Stern–Volmer plots of the data showed similarity, and a recovery of the luminescence was not possible. This, along with the evidence that a different MOF formation was observed (Figure 21) and no overlap of the absorption of these ions with the excitation of the LnBDC was observed (Figure 21), provides a reason to exclude as an option mechanism (iv) mentioned before but confirms that the destruction of the initial MOF (iii) is the most important effect.

In the case of Fe(III) and CrO_4_^2−/^Cr_2_O_7_^2^− quenching, we have to consider that the luminescence of Ln-MOFs mainly suffers from the “antenna effect”, i.e., the ligands efficiently absorb and transfer light to the luminescent central lanthanide ions. As shown in Figure 21, the UV–VIS absorption spectra of Fe(III) and CrO_4_^2−/^Cr_2_O_7_^2−^ emerged as strong absorption bands in the range of 200–350 nm for Fe(III) and 260–400 nm for CrO_4_^2−/^Cr_2_O_7_^2−^, while the excitation of LnBDC was at 320 nm. These facts imply that Fe(III) and CrO_4_^2−/^Cr_2_O_7_^2^ could drastically absorb the energy of the excitation, reducing the efficiency of energy transfer from the ligand to the lanthanide ions and, by that, definitely induce the luminescence-quenching effect. All these suggest a Förster resonance energy transfer again.

## 3. Materials and Methods

### 3.1. Materials Used

All the chemicals for the experiments were analytical grade. Terephthalic acid was procured from Thermo Scientific™, Waltham, MA, USA. LnCl_3_∙nH_2_O was synthesized from Ln_2_O_3_ (Ln = Sm, Eu, Dy) and Tb_4_O_7_ (Fluka p.a., Shanghai, China) by dissolving in dilute HCl, followed by crystallization and recrystallizations. Stock solutions of salts (CHEM-LAB, Belgium) were used for sensor analysis.

### 3.2. Synthesis of the Powder Samples SmBDC, EuBDC, TbBDC, and DyBDC

Powder samples with the composition ([Ln_2_(BDC)_3_(H_2_O)_4_]_∞_ (Ln = Sm, Eu, Tb, Dy) were synthesized under solvothermal conditions. Corresponding LnCl_3_·nH_2_O (2.0 mmol) and H_2_BCD (3.0 mmol) were dissolved in 48 mL of DMF (0.62 mol). Next, the solution was placed in a 100 mL Teflon-lined stainless-steel reactor and kept at 130 °C for 5 days. After natural cooling to room temperature, the precipitate was washed twice with DMF and three times with water and dried at 80 °C.

Powder XRD confirmed that all four samples were isostructural (see Section 2.2). Elemental analysis of the sample with the empirical formula [Ln_2_(BDC)_3_(H_2_O)_4_]_∞_ showed the found/calculated percentage (%) for EuBDC: C 33.02/33.10, H 2.09/2.32; TbBDC: C 33.04/32.67, H 2.02/2.28; SmBDC: C 32.94/33.32, H 2.11/2.33; and DyBDC: C 32.04/32.41, H 2.03/2.27.

IR analysis (see Figure S11) additionally confirmed that the samples were isostructural, and there was a coordination to H_2_BDC. The vibrations assignment of the free terephthalic acid was done according to [58].

### 3.3. Single-Crystal Growth of DyBDC-SC

A single crystal of the powdered sample with the composition [Ln_2_(BDC)_3_(H_2_O)_4_]_∞_ was obtained under solvothermal conditions. DyCl_3_·nH_2_O (0.1 mmol) and H_2_BCD (0.05 mmol) were dissolved in 11 mL of acetonitrile/water mixture (1:1.75 volume ratio), and 0.3 mL of 1 mol/L of HCl was added to the solution. The mixture was placed in a 20 mL Teflon-lined stainless-steel reactor and kept at 140 °C for 72 h, with a 15 °C/h step of increasing temperature and a 5 °C/h step of cooling. After cool-down, a small number of crystals were found suitable to be measured with single-crystal diffraction. It was found that the structure solved corresponded to the one reported first in [9].

### 3.4. Characterization

The data set was collected using a Bruker D8 Venture diffractometer with a micro-focus sealed tube and a Photon II detector. Monochromated MoKα radiation (λ = 0.71073 Å) was used. Data were collected at 133(2) K and corrected for absorption effects using the multi-scan method. The structure was solved by direct methods using SHELXT [59]) and was refined by full-matrix least squares calculations on F2 (SHELXL2018 [60]) in the graphical user interface Shelxle [61]. Powder XRD measurements were performed on a PANalytical Empyrean diffractometer (Malvern PANalytical Empyrean, Almelo, the Netherlands) with a PIXcel 3D detector and CuKα radiation (λ = 0.1542 nm). Microstructural information was extracted by full-profile Rietveld refinement using the GSAS II crystallography software package [62]. Infrared spectral analysis was carried out on an FTIR Nicolet 6700 (Thermo Scientific) in KBr pellets. Scanning electron microscopy (Hitachi TM4000; Krefeld, Germany) was used to observe the morphology of the obtained materials. Lifetime measurements and photoluminescence measurements of the complexes were conducted on a Cary Eclipse spectrometer with a xenon lamp as the excitation source. For powder samples, a powder cell with a Cary Eclipse Solid Sample Holder was used, and for suspension measurements, 1 cm fluorescent quartz cuvettes were used. The textural characteristics of the samples were determined by low-temperature nitrogen adsorption. The adsorption and desorption isotherms of nitrogen at −196 °C were determined in the pressure range of p/p_0_ = 0.001–1 using “AUTOSORB iQ-MP/AG” (Anton Paar GmbH, Graz, Austria). Before every measurement, the samples were degassed at 250 °C for 16 h. pH measurements were performed using the XS sensor pH 8+ DHS kit BASIC with a micro P electrode. A Q55 Sonicator probe was used for homogenization of the suspensions.

### 3.5. Fluorescent Measurements in Suspension

#### 3.5.1. Suspension Concentration

The suspensions for all sensing experiments were prepared using the as-prepared MOFs. A stock aqueous suspension with a concentration of 1.0 mg/mL was prepared. The suspension was treated with a Q55 Sonicator probe for several minutes. Next, by dilution, 4 suspensions with concentrations of 0.7, 0.5, 0.3, and 0.1 mg/mL were prepared, followed by sonication and measurement of their luminescence under the same conditions (emission spectra: λex = 320 nm, ex/em slit: 5/2.5 nm, data interval: 1.0 nm, average time: 0.10 s, PMT voltage: 800 V).

For both EuBDC and TbBDC, a 0.5 mg/mL concentration was chosen, demonstrating the best stability and excluding self-quenching effects (Figure S4).

#### 3.5.2. Procedure for pH Measurements of the Suspensions

For both samples, 10 vials with different pH values were prepared. A solution of KCl (0.1 mol/L) was used to keep the ionic strength constant. HCl and KOH (0.1 mol/L) were added to the KCl solution to adjust the pH value in steps of around 1.0. When the desired pH value was reached, a certain volume from the solution was added to the preweighed sample vial so that the concentration of the suspension was adjusted to 0.5 mg/mL. All samples were sonicated and measured under the same conditions (emission spectra: λex = 320 nm, ex/em slit: 5/2.5 nm, data interval: 1.0 nm, average time: 0.10 s, PMT voltage: 800 V).

#### 3.5.3. Procedure for Cation and Anion Sensing Experiments

To explore the behavior of TbBDC and EuBDC in the presence of different cations and anions, 1 mg/mL of a stock aqueous suspension was prepared. Stock solutions of cations and anions with a concentration of 1 × 10^−2^ mol/L were used. Cations were nitrate salts, and anions were potassium and sodium salts. Briefly, 1.0 mL of the starting suspension was mixed with 0.8 mL of water and 0.2 mL of the stock solution of cations and anions, so the resulting concentration of the suspensions was 0.5 mg/mL, and the concentration of cations and anions was 1 × 10^−3^ mol/L. All samples were then measured with a pH meter (Table S4) to confirm we were in the working pH range of the sensors.

#### 3.5.4. Procedure for Stern–Volmer Data Collections and Data Management

Briefly, suspensions with a starting concentration of 0.5 mg/mL were prepared in two parallel cuvettes, and each was measured five times to determine the standard deviation of the blank sample. Next, to one of them was added the exact volume of the stock solution of the chosen ion, and to the other was added the same volume of water. Each time, both samples were measured three times. Baseline correction was performed, and for each concentration, the average value of the intensity from the parallel measurements was taken (for TbBDC at λ = 547 nm and for EuBDC at λ = 616 nm). Since dilution with water during the experiment in the second cuvette did not lead to a significant decrease in the intensity of the blank sample, we used an average value of all measurements of the blank sample for I_0_.

When working with luminescent sensors, we need to apply the Stern–Volmer equation. The simplest case is to observe the linear dependence of Stern–Volmer data, but it is not an uncommon thing to have nonlinear dependence, suggesting a different quenching mechanism or more than one quenching mechanism taking part. As reported by W. Laws and P. Contino [63], when linear dependence is observed, we may have the dynamic quenching mechanism described by Equation (1):

I_0_/I = 1 + K_sv_[Q] (1)

I_0_ is the intensity in the absence of a quencher;

I is the intensity in the presence of a quencher;

K_sv_ is the Stern–Volmer constant corresponding to the dynamic quenching constant K_D_;

[Q] is the concentration of the quencher.

Similarly, Equation (2), with linear dependence, can describe static quenching, where quenching occurs as a result of the formation of a nonfluorescent ground-state complex between the fluorophore and the quencher.

I_0_/I = 1 + K_S_[Q] (2)

Only here, K_S_ is the association constant. Static quenching removes a fraction of the fluorophores from observation. The complexed fluorophores are nonfluorescent, and the only observed fluorescence is from the uncomplexed fluorophores. The uncomplexed fraction is unperturbed, and hence, the lifetime is τ_0_. Therefore, for static quenching, τ_0_/τ = 1. In contrast, for dynamic quenching, τ_0_/τ follows the same linear dependence as I_0_/I.

When both static quenching and dynamic quenching are combined, we observe both quenching by collisions and quenching by complex formation with the same quencher. The Stern–Volmer equation is a second-order polynomial curve described by Equation (3):

I_0_/I = K_D_K_S_[Q]^2^ + (K_D_ + K_S_) [Q] + 1 (3)

The dynamic portion of the observed quenching can be determined by lifetime measurements, i.e., we modify Equation (3) to allow a graphical separation of K_S_ and K_D_ [63,64,65,66].

Kapp = (I_0_/I−1)/[Q] = (K_D_ + K_S_) + K_D_.K_S_[Q] (4)

K_S_^2^ – K_S_I + S = 0 (5)

where I is the intercept and S is the slope of the graphical representation of Kapp to the concentration of the quencher.

There is another type of static quenching in which the quencher does not actually form a ground-state complex. Instead, it seems that the apparent static component is due to the quencher being adjacent to the fluorophore at the moment of excitation. These closely spaced fluorophore–quencher pairs are immediately quenched and thus appear to be dark complexes. This quenching is called “sphere of action”, within which the probability of quenching is unity. The modified form of the Stern–Volmer equation that describes this situation is Equation (5):

I_0_/I = (1 + K_sv_[Q])e^V[Q]^ (6)

where V is the volume of the sphere. When the fluorophore and the quencher are this close, there exists a high probability that quenching will occur before these molecules diffuse apart. As the quencher concentration increases, the probability increases that the quencher is within the first solvent shell of the fluorophore at the moment of excitation [63,64].

Several studies on sensors with exponential curves have used the modified Equation (6):

I_0_/I = Ae^k[Q]^ + B (7)

They have calculated the Stern–Volmer constant as a product of constant A and constant k in Equation (7) [67,68,69,70,71].

## 4. Conclusions

The Ln-MOFs (Ln = Sm, Eu, Tb, Dy) based on terephthalic acid were synthesized by the solvothermal method, and the characterization by XRD proved that isostructural samples were obtained. The detailed investigation of the crystal parameters of the samples performed can be considered an extension of the data on the crystal structures of the Ln-MOFs. The microstructural information received by Rietveld refinement displayed the influence of the ionic radius of Ln(III) both on the unit cell parameters and the crystallites size and on the defective structure.

Luminescent properties evidenced both Eu- and Tb-MOFs as candidates for further sensor investigations. The systematic study of the stability in water and in an environment with different acidity levels confirmed the potential of the investigated samples as sensors. The complex character of the quenching process involving different ions was revealed in which dynamic quenching and static quenching operate simultaneously. Based on the luminescence-quenching effect with a fast response, the Tb-MOF can be applied to identify both Fe(III) and Cr(VI) ions in water solutions.

## Figures and Tables

**Table 1:** Lifetimes in the solid state and in a water suspension.

Sample	Lifetime in the Solid State, µs	Lifetime in a Water Suspension, µs
SmBDC	UDL ^1^	UDL ^1^
EuBDC	390 ± 3	389 ± 3
TbBDC	901 ± 1	885 ± 1
DyBDC	UDL^1^	UDL^1^

^1^ UDL—under detection limit.

## Data Availability

Crystallographic data have been deposited at the Cambridge Crystallographic Data Center under the reference number 2366456.

## Supplementary Materials

The following supporting information can be downloaded at https://www.mdpi.com/article/10.3390/molecules29153713/s1: Figure S1. Rietveld plots for (a) SmBDC, (b) EuBDC, (c) TbBDC and (d) DyBDC.; Figure S2. Comparison of solid-state emission coming from different Ln(III) ions’ normalized spectra: (a) SmBDC, (b) EuBDC, (c) TbBDC, and (d) DyBDC; Figure S3. Nitrogen adsorption–desorption isotherms of EuBDC and TbBDC; Figure S4. Luminescence spectra of different suspensions, (a) EuBDC and (b) TbBDC, taken under the same conditions with λex = 320 nm; Figure S5. Luminescence spectra of (a) EuBDC and (b) TbBDC in an acetone suspension and after removal of acetone in a water suspension, taken under the same conditions with λex = 320 nm; Figure S6. XRD patterns of the TbBDC sample. Suspensions with pH 1.50 and 11.90 are left overnight. Next, they are centrifuged and washed several times with water until a neutral reaction and dried; Figure S7. XRD patterns of the samples after full quenching with Ag(I) compared to the DyBDC-SC and the single crystal of AgBDC; Figure S8. TbBDC linear dependency interval for Fe(III); Figure S9. Graphical separation of KS and KD for TbBDC with Cr(VI); Figure S10. TbBDC linear dependency interval for Cr(VI); Figure S11. IR spectra in the region of (a) 2000–400 cm^−1^ and (b) 4000–1750 cm^−1^ of Ln-MOFs compared to a free linker. Table S1. Experimental details of single-crystal diffraction; Table S2. Unit cell parameters, unit cell volume and microstructural characteristics of the LnBDC obtained by the Rietveld refinements. Table S3. Textural properties of EuBDC and TbBDC. Table S4. pH values measured in the suspensions. Table S5. Summary of Stern-Volmer data.
