# Supplementary material for: Investigation of the Sensing Properties of Lanthanoid Metal–Organic Frameworks (Ln-MOFs) with Terephthalic Acid
Source: Molecules. 2024 Aug 5;29(15):3713. doi: 10.3390/molecules29153713 (PMC11314416; doi:10.3390/molecules29153713)
Supplement: Supplementary file 1 [file molecules-29-03713-s001.zip › molecules-3106877-supplementary.pdf]

Investigation of the Sensing Properties of Lanthanoid Metal–Organic Frameworks (Ln-MOFs) with Terephthalic Acid

Denitsa Elenkova [1,*], Yana Dimitrova [1], Martin Tsvetkov [1], Bernd Morgenstern [2], Maria Milanova [1], Dimitar Todorovsky [1] and Joana Zaharieva [1,*]

[1] Faculty of Chemistry and Pharmacy, Sofia University, 1164 Sofia, Bulgaria; yanadim123@gmail.com (Y.D.); nhmt@chem.uni-sofia.bg (M.T.); nhmm@chem.uni-sofia.bg (M.M.); dtodorovsky@yahoo.com (D.T.)

[2] Inorganic Solid State Chemistry, Saarland University, Campus Geb. C4 1, 66123 Saarbrücken, Germany; bernd.morgenstern@uni-saarland.de

* Correspondence: nhde@chem.uni-sofia.bg (D.E.); nhjz@chem.uni-sofia.bg (J.Z.)

Content

Figure S1. Rietveld plots for (a) SmBDC, (b) EuBDC, (c) TbBDC and (d) DyBDC.

Figure S2. Comparison of solid-state emission coming from different Ln(III) ions' normalized spectra: (a) SmBDC, (b) EuBDC, (c) TbBDC, and (d) DyBDC.

Figure S3. Nitrogen adsorption – desorption isotherms of EuBDC and TbBDC.

Figure S4. Luminescence spectra of different suspensions, (a) EuBDC and (b) TbBDC, taken under the same conditions with λex = 320 nm.

Figure S5. Luminescence spectra of (a) EuBDC and (b) TbBDC in an acetone suspension and after removal of acetone in a water suspension, taken under the same conditions with λex = 320 nm.

Figure S6. XRD patterns of the TbBDC sample. Suspensions with pH 1.50 and 11.90 are left overnight. Next, they are centrifuged and washed several times with water until a neutral reaction and dried.

Figure S7. XRD patterns of the samples after full quenching with Ag(I) compared to the DyBDC-SC and the single crystal of AgBDC.

Figure S8. TbBDC linear dependency interval for Fe(III).

Figure S9. Graphical separation of K_S and K_D for TbBDC with Cr(VI).

Figure S10. TbBDC linear dependency interval for Cr(VI).

Figure S11. IR spectra in the region of (a) 2000-400cm^{-1} and (b) 4000-1750cm^{-1} of Ln-MOFs compared to a free linker

Table S1. Experimental details of the single crystal diffraction.

Table S2. Unit cell parameters, unit cell volume and microstructural characteristics of the LnBDC obtained by the Rietveld refinements.

Table S3. Textural properties of EuBDC and TbBDC.

Table S4. pH values measured in the suspensions.

Table S5. Summary of Stern–Volmer data.

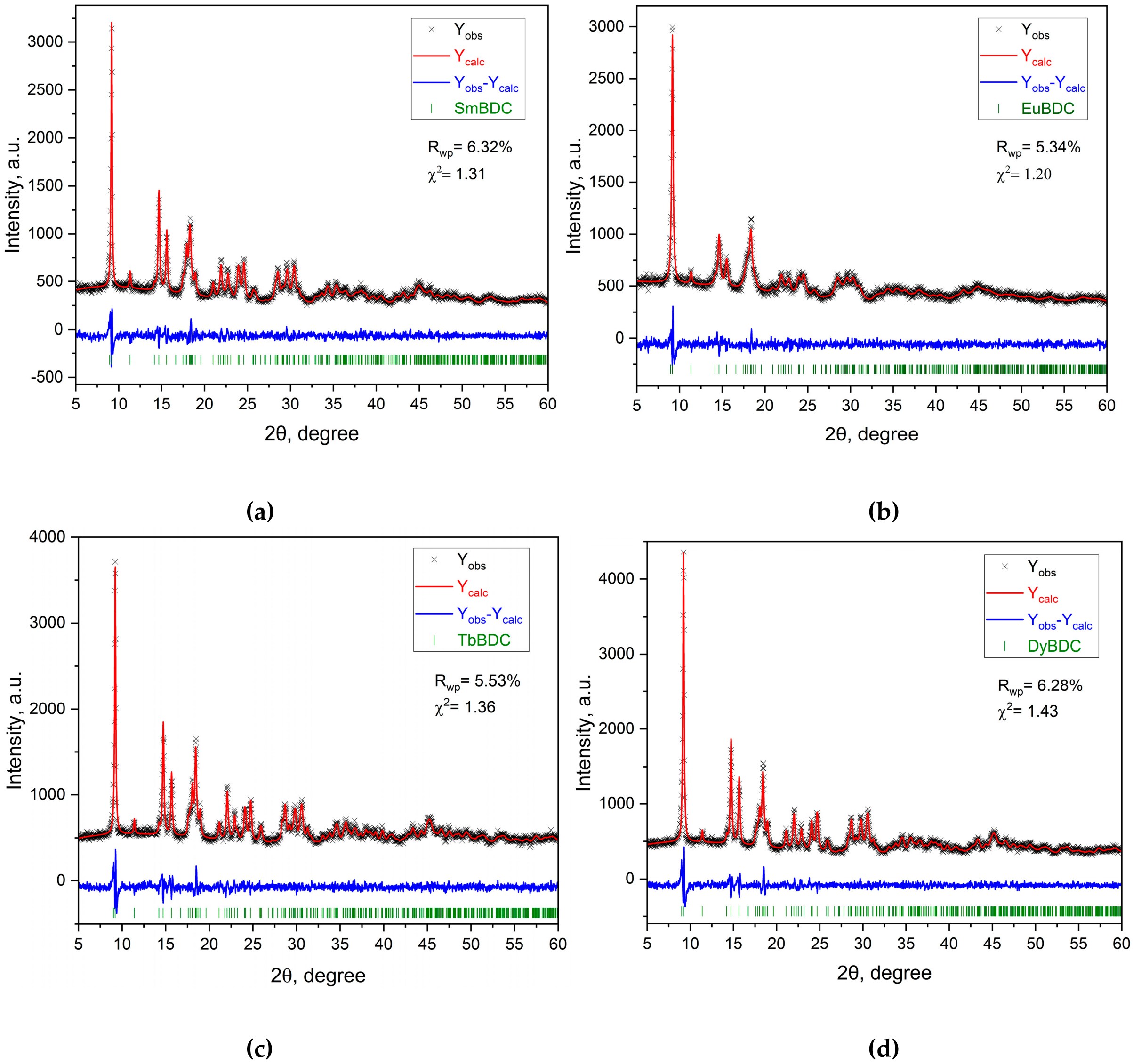

Figure S1. Rietveld plots for (a) SmBDC, (b) EuBDC, (c) TbBDC and (d) DyBDC.

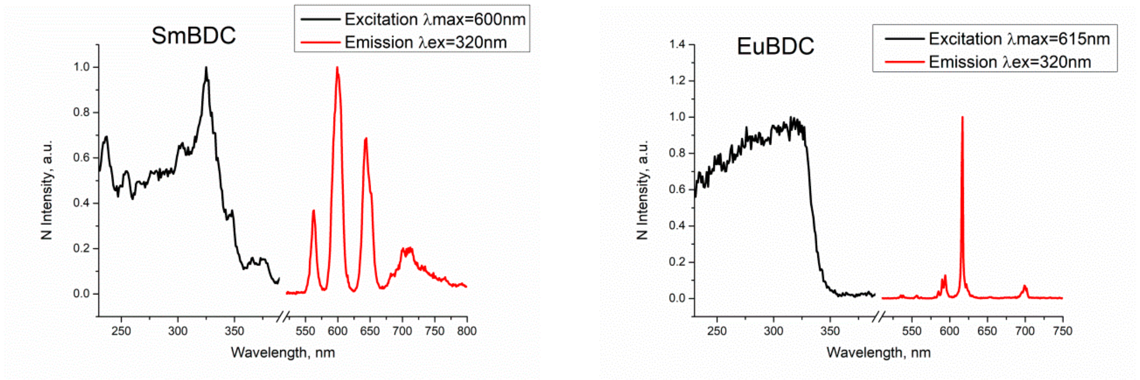

(a) **(b)**

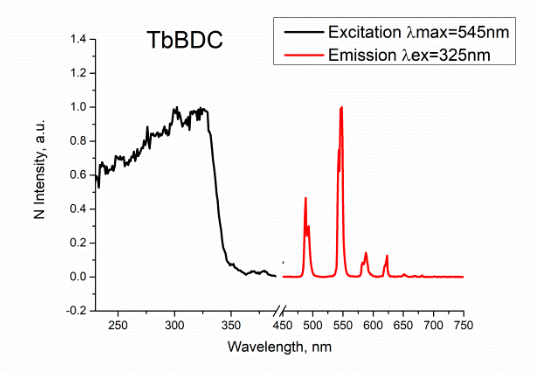

(c)

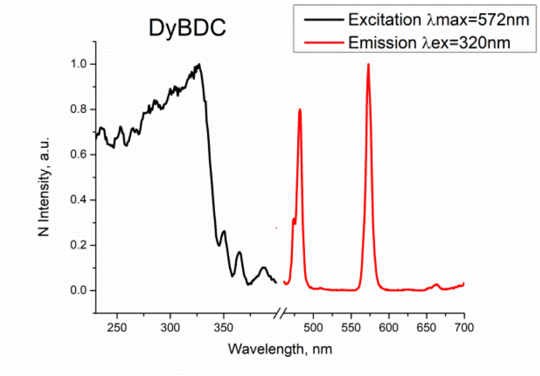

(d)

Figure S2. Comparison of solid-state emission coming from different Ln(III) ions' normalized spectra: (a) SmBDC, (b) EuBDC, (c) TbBDC, and (d) DyBDC.

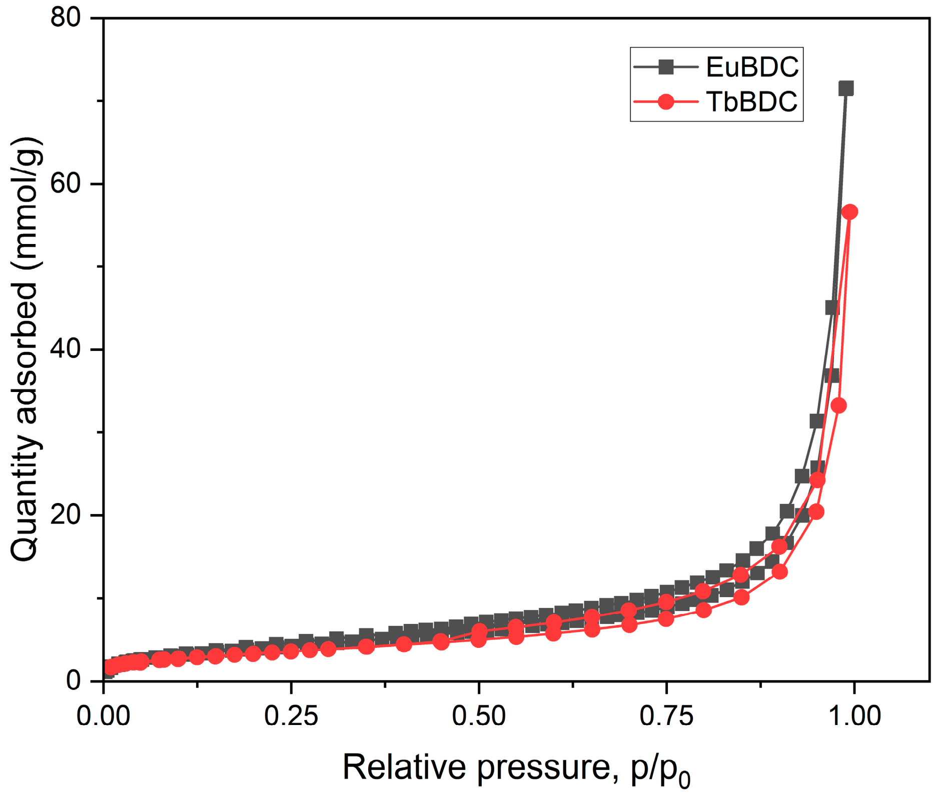

Figure S3. Nitrogen adsorption – desorption isotherms of EuBDC and TbBDC.

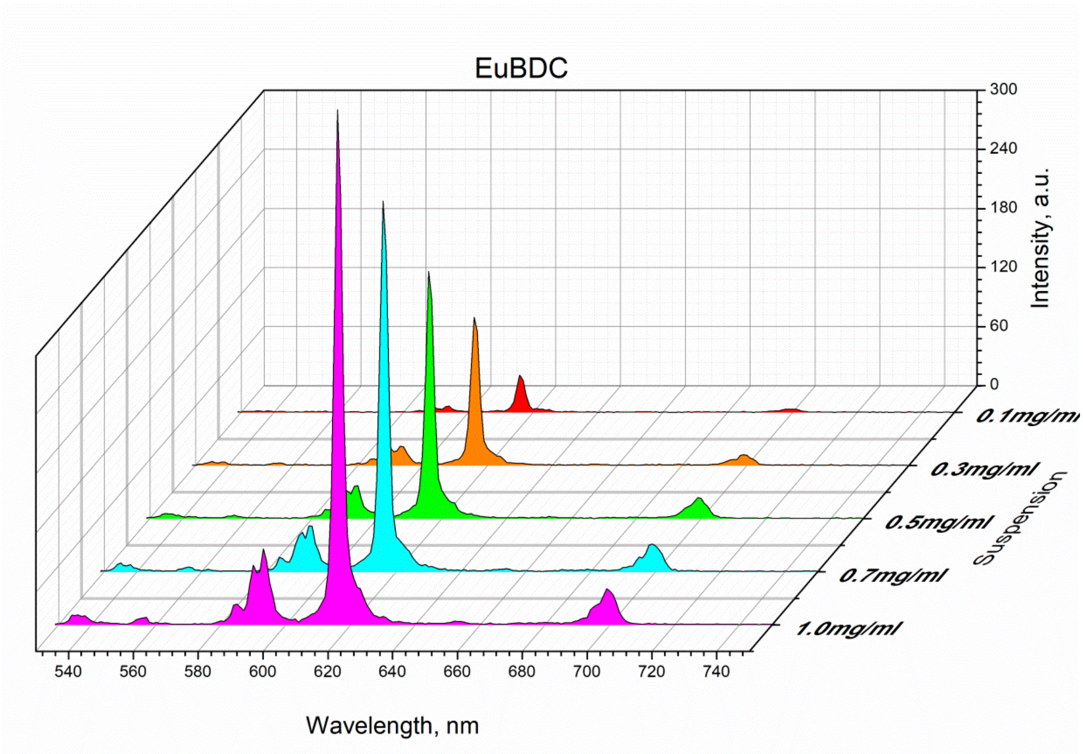

(a)

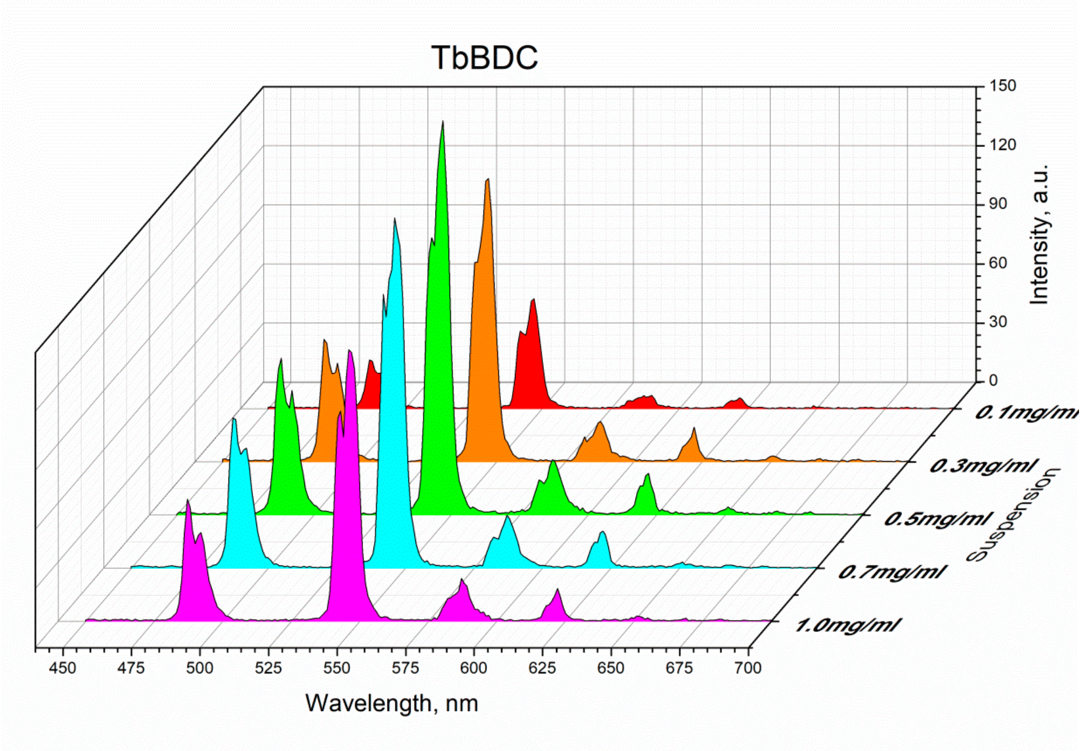

(b)

Figure S4. Luminescence spectra of different suspensions, (a) EuBDC and (b) TbBDC, taken under the same conditions with $\lambda ex = 320$ nm.

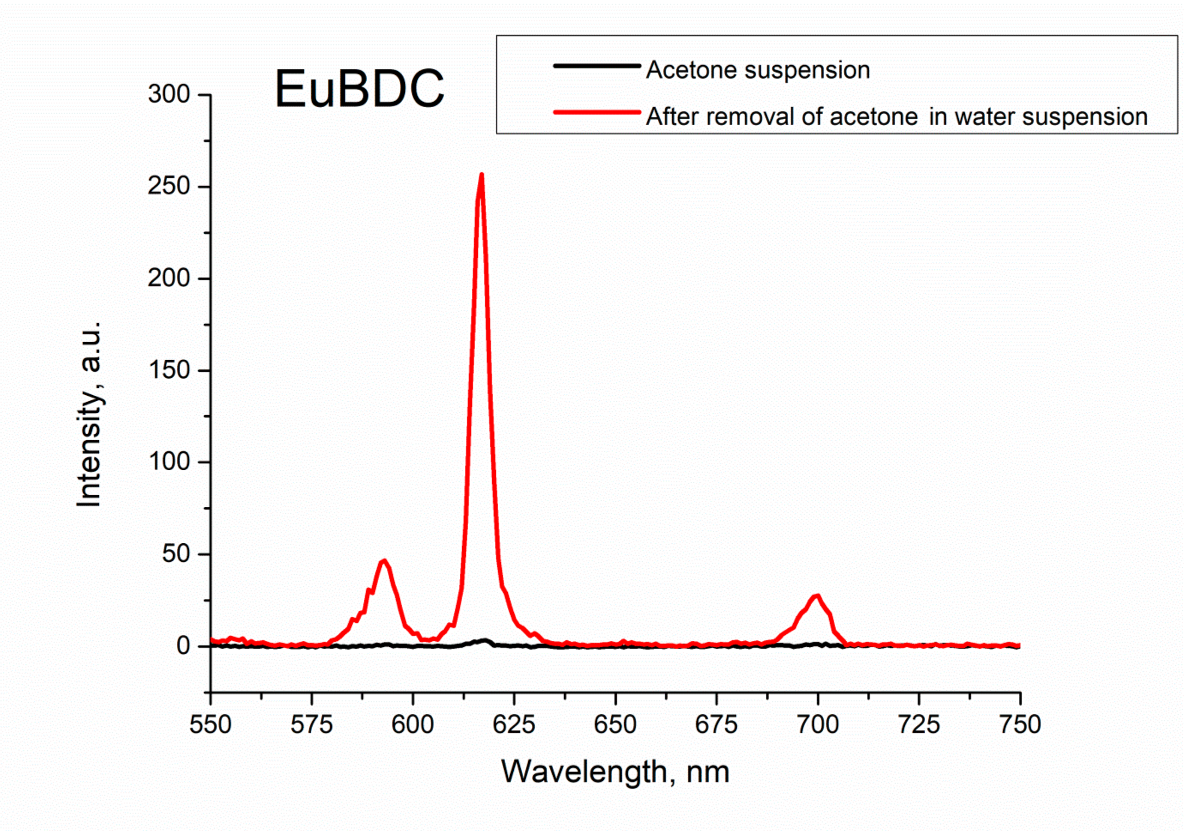

EuBDC
Acetone suspension
After removal of acetone in water suspension
Intensity, a.u.
Wavelength, nm
300
250
200
150
100
50
0
550
575
600
625
650
675
700
725
750

(a)

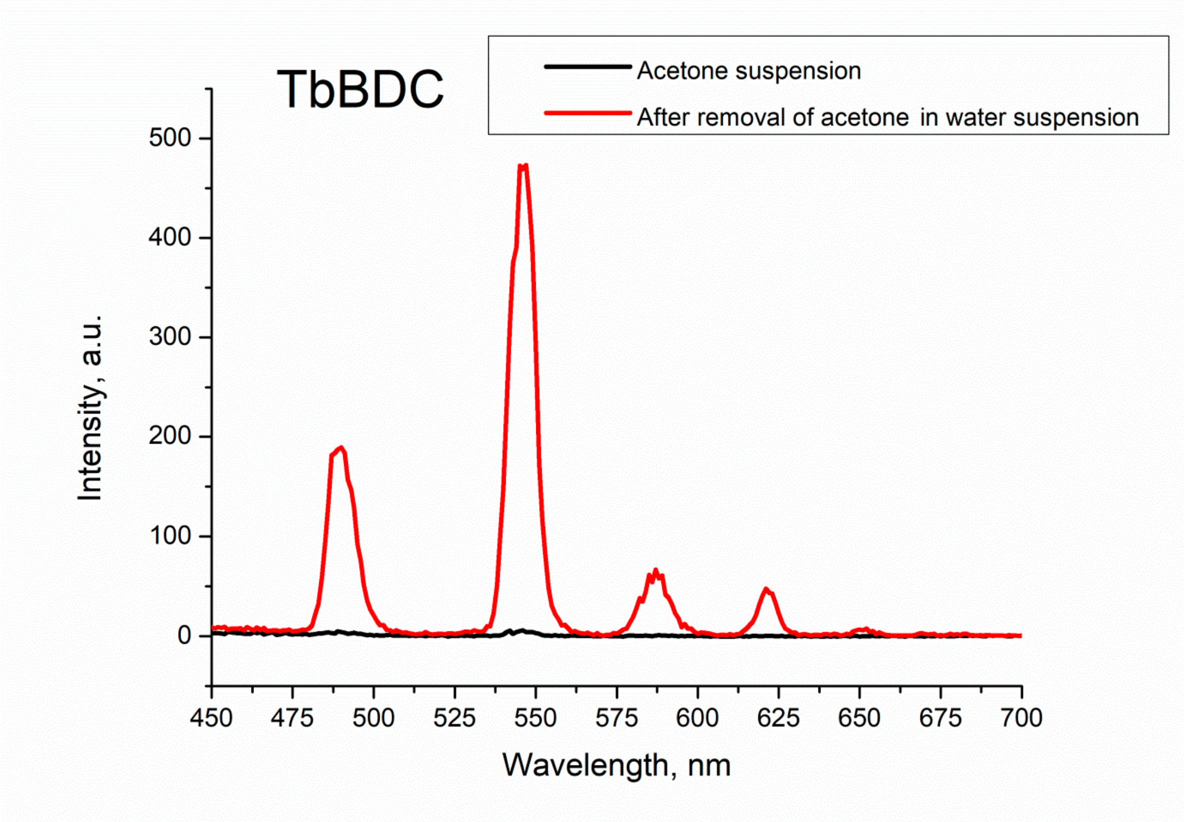

TbBDC
Acetone suspension
After removal of acetone in water suspension
Intensity, a.u.
Wavelength, nm
500
400
300
200
100
0
450
475
500
525
550
575
600
625
650
675
700

(b)

Figure S5. Luminescence spectra of (a) EuBDC and (b) TbBDC in an acetone suspension and after removal of acetone in a water suspension, taken under the same conditions with λex = 320 nm.

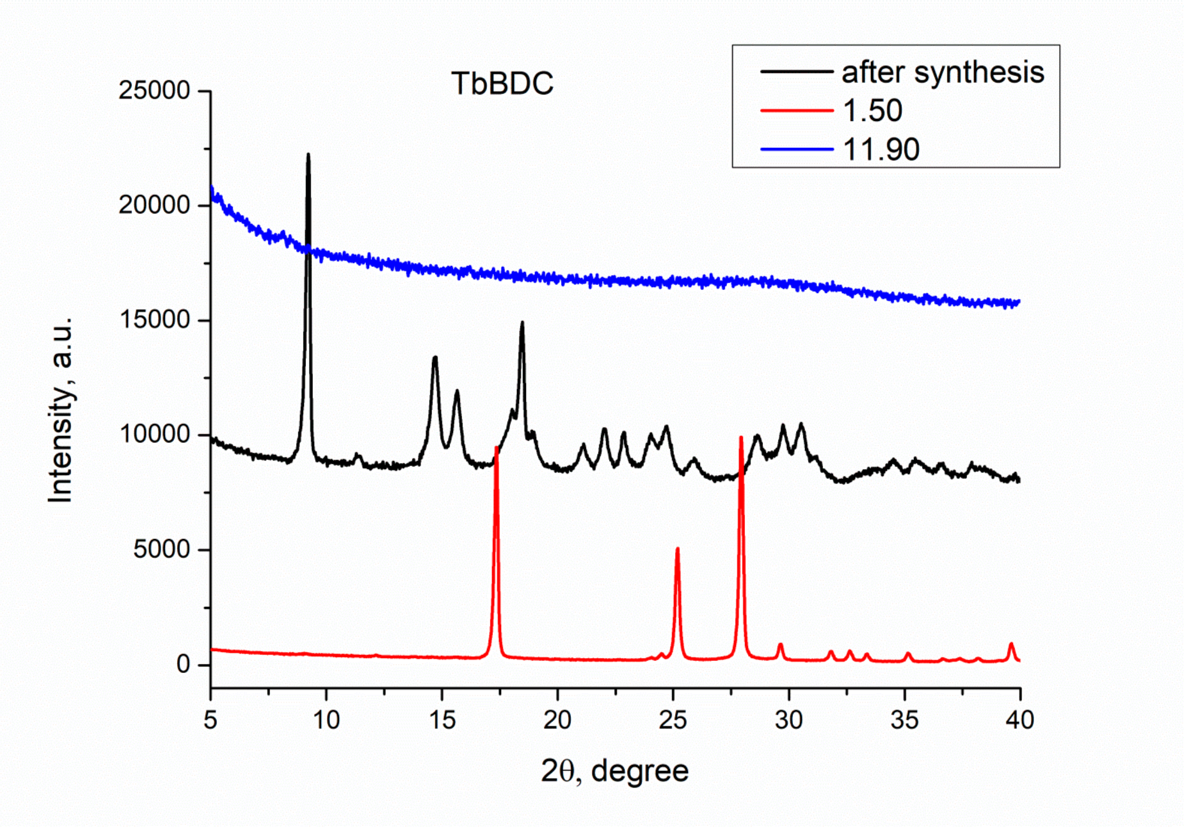

Figure S6. XRD patterns of the TbBDC sample. Suspensions with pH 1.50 and 11.90 are left overnight. Next, they are centrifuged and washed several times with water until a neutral reaction and dried.

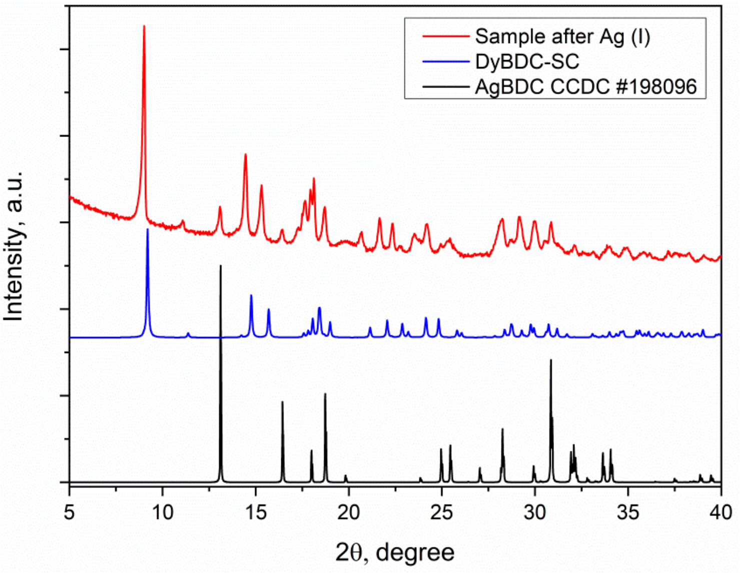

Figure S7. XRD patterns of the samples after full quenching with Ag(I) compared to the DyBDC-SC and the single crystal of AgBDC.

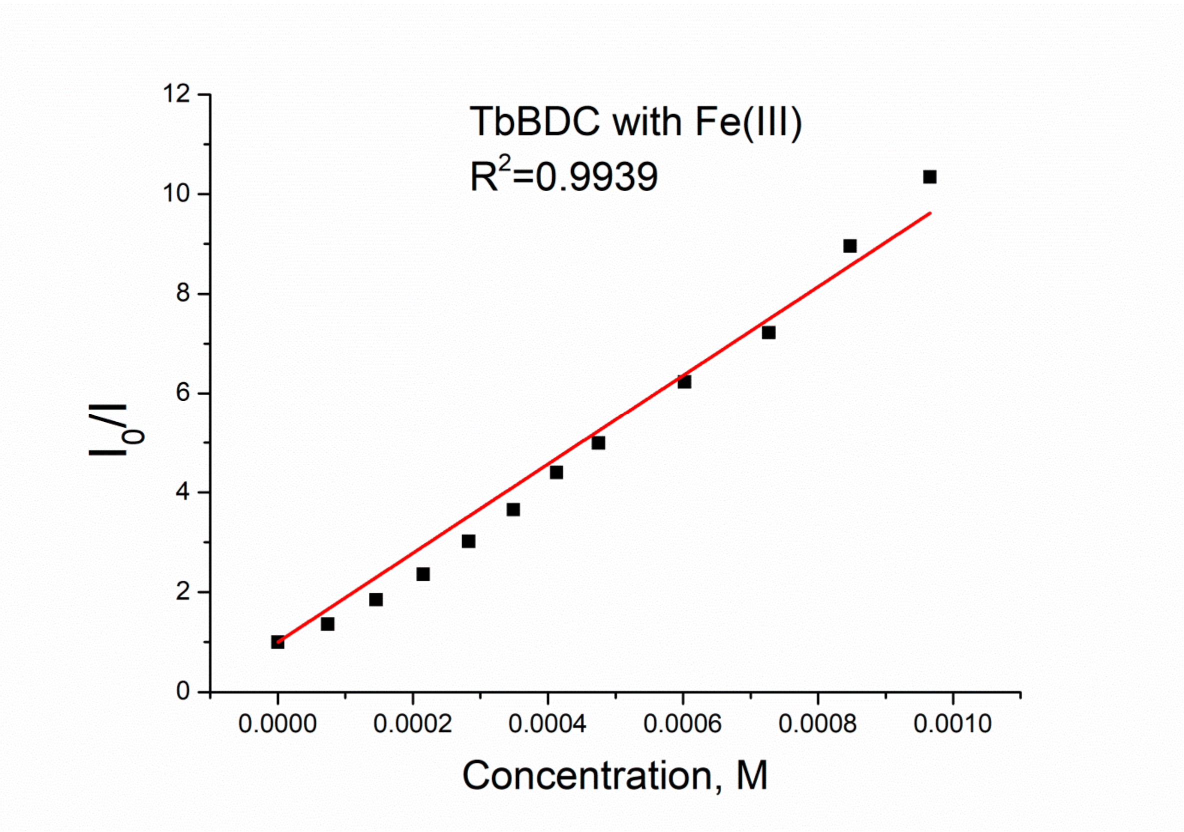

Figure S8. TbBDC linear dependency interval for Fe(III).

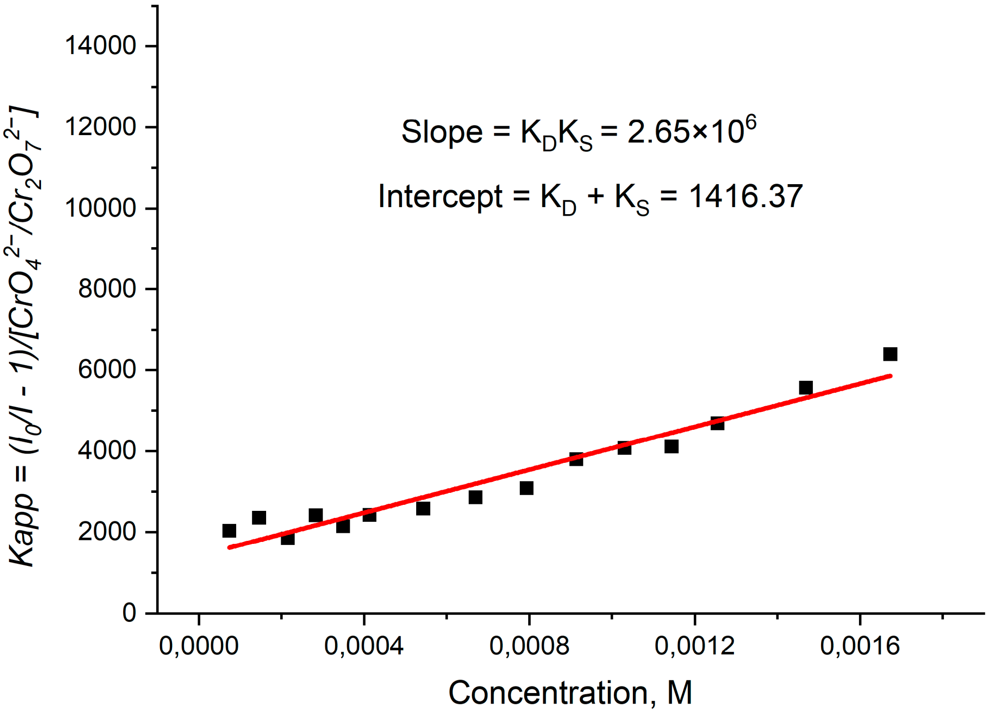

Figure S9. Graphical separation of K_S and K_D for TbBDC with Cr(VI).

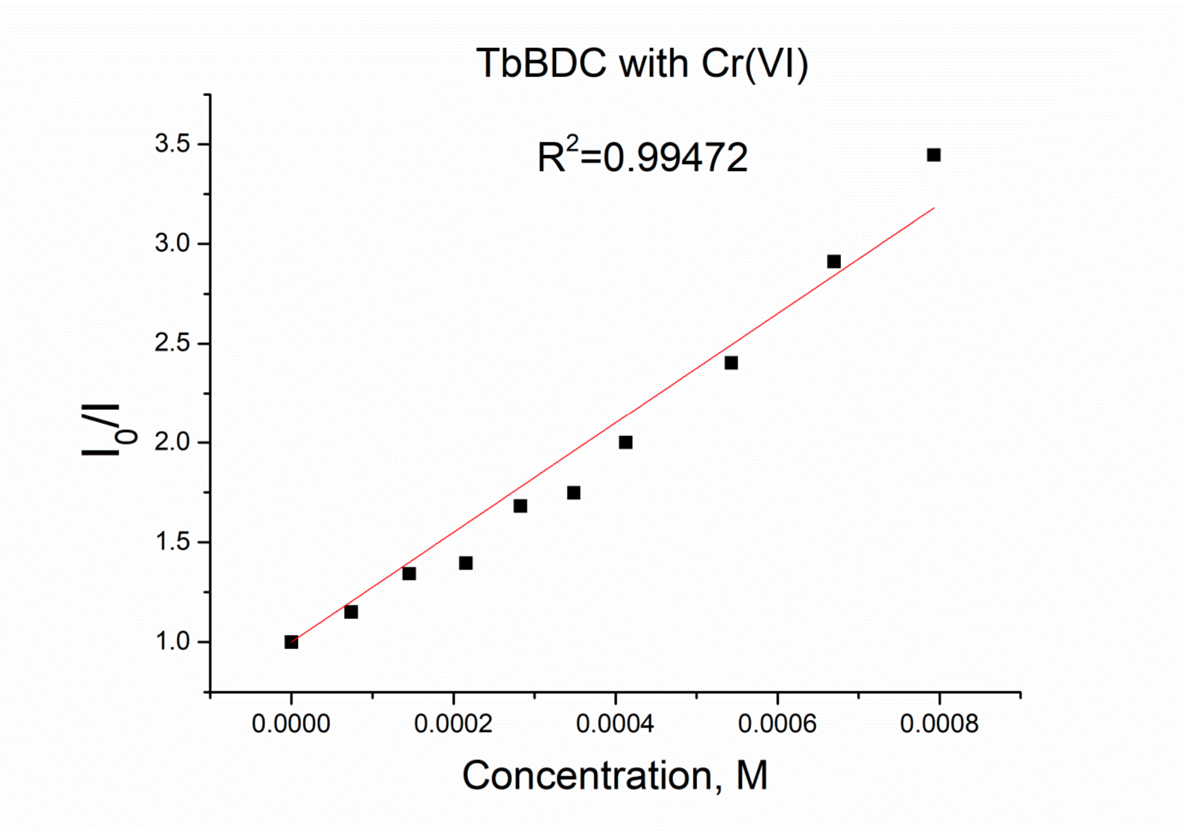

Figure S10. TbBDC a linear dependency interval for Cr(VI)

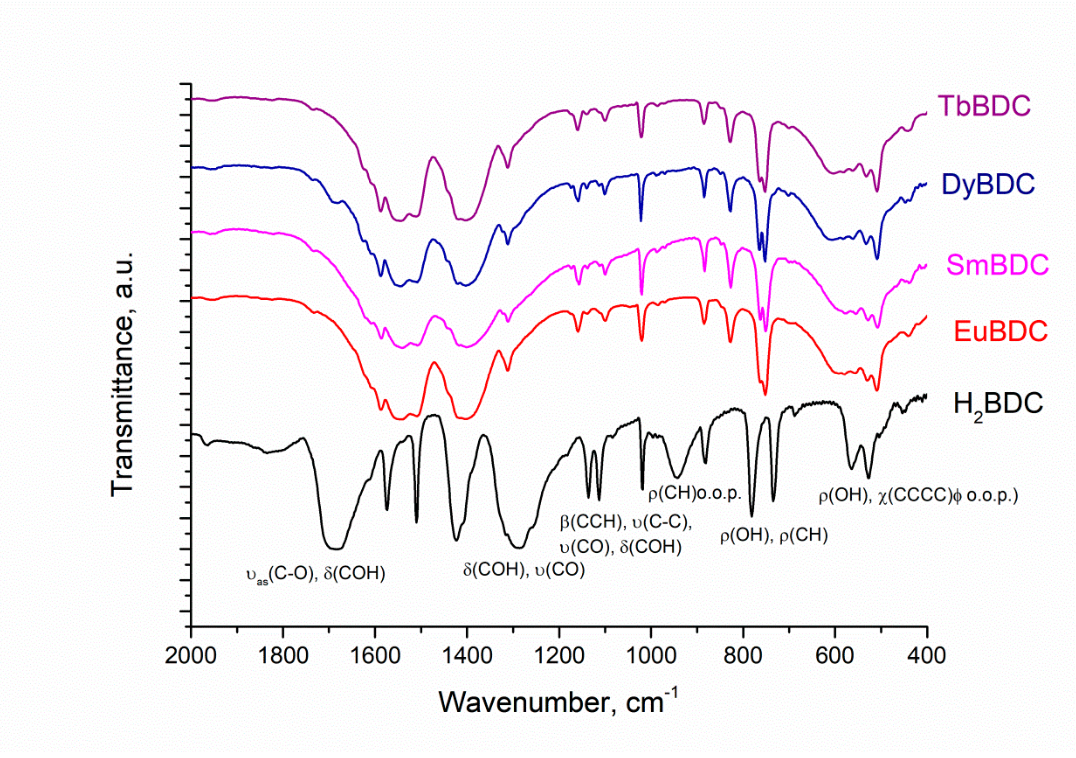

(a)

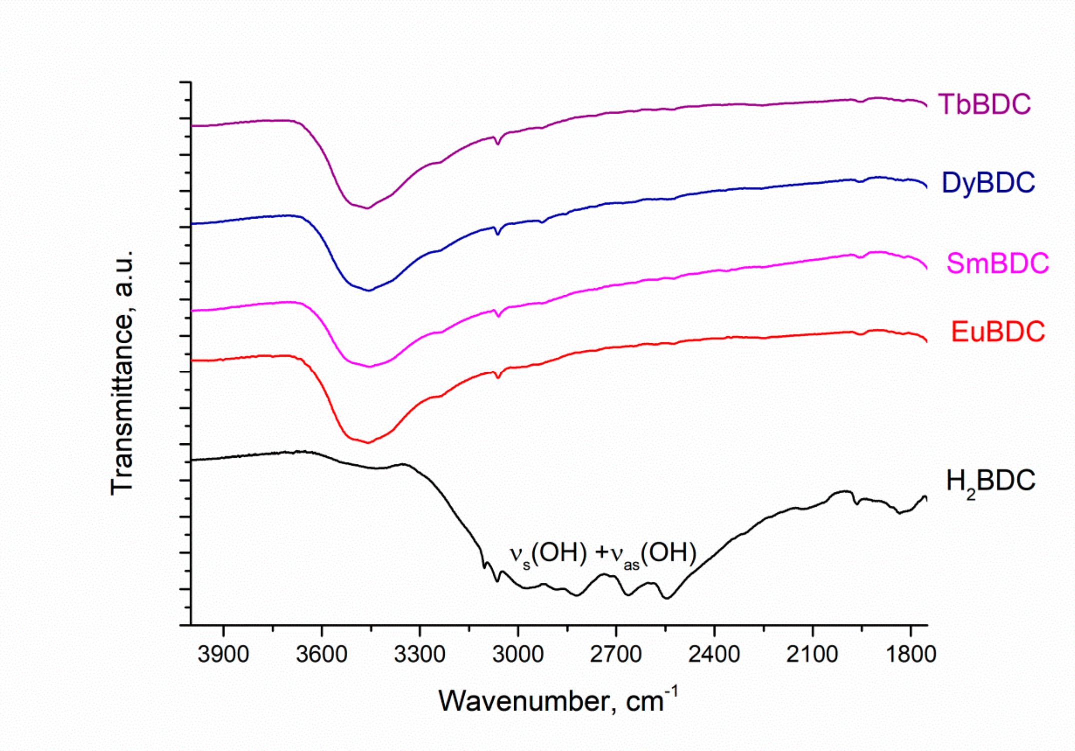

(b)

Figure S11. IR spectra in the region of (a) 2000-400cm^{-1} and (b) 4000-1750cm^{-1} of Ln-MOFs compared to a free linker .

The vibrations assignment of the free terephthalic acid was done according to [58]. It is clearly seen that when complexation occurs all of the characteristic bands for the carboxylic group disappear which is an indication that the reaction is going on between the carboxylic group and the Ln(III) ion. Furthermore, a clear band around 3500-3600 cm^{-1} due to inclusion of water molecules in the crystal structure is detected. As expected, significant differences in the FT-IR spectra of the different Ln-MOFs are not observed, indicating additionally for their identical chemical composition and structure.

Table S1. Experimental details of the single crystal diffraction.

**Chemical formula**	$C_{12}H_{10}O_8Dy$
**CCDC number**	2366456
**Molecular weight (g/mol)**	444.70
**Crystal system, Space group**	Triclinic *P-1*
**Temperature (K)**	133(2)
a, b, c (Å)	6.1294(9)

	10.0465(15)<br>10.0608(15)
***α, β, γ* (°)**	102.190(5)<br>91.180(5)<br>101.475(5)
**Unit cell volume Å^3**	592.19(15)
**Z**	2
**Radiation type**	Mo *Kα*
**μ, (mm^{-1})**	6.351
**Crystal size (mm)**	0.150 x 0.100 x 0.020
**Data collection**	
**Diffractometer**	Bruker D8 Venture
**Absorption correction**	multi-scan<br>*TWINABS*, Version 2012/1<br>[30]
**T_{min}, T_{max}**	0.5607, 0.7456
**No. of measured, independent and observed [I > 2σ(I)] reflections**	4632, 4632, 4544
**(sin θ/λ)max, (Å−1)**	0.659
**Refinement**	
**R[F^2 > 2σ(F^2)], wR(F^2), S**	0.0225, 0.0636, 1.086
**No. of reflections**	4632
**No. of restrains**	16
**No. of parameters**	203
**H-atom treatment**	mixed

Δρmax, Δρmin (e Å−3)	0.618, -0.923

Table S2. Unit cell parameters, unit cell volume and microstructural characteristics of the LnBDC obtained by the Rietveld refinements.

Sample	Unit cell parameters	Unit cell volume, Å^3	Crystallites size, nm	Microstrains, x10^{-2} a.u.
**SmBDC**	a= 6.2116(7)<br>b= 10.1143(3)<br>c= 10.1313(9)<br>α= 101.89(9)<br>β= 91.55(6)<br>γ= 101.79(3)	608.1(5)	180.1(11)	1.9(1)
**EuBDC**	a= 6.1847(2)<br>b= 10.1186(3)<br>c= 10.1608(7)<br>α= 102.14(9)<br>β= 91.45(6)<br>γ= 101.59(3)	607.5(4)	173.0(2)	1.8(2)
**TbBDC**	a= 6.1620(2)<br>b= 10.0603(10)<br>c= 10.0910(7)<br>α= 102.03(3)<br>β= 91.44(5)<br>γ= 101.43(4)	598.2(3)	142.3(1)	1.4(4)
**DyBDC**	a= 6.1570(2)<br>b= 10.0414(9)<br>c= 10.0676(12)<br>α= 102.02(3)<br>β= 91.49(7)<br>γ= 101.35(9)	595.4(3)	139.4(10)	1.6(1)

Table S3. Textural properties of EuBDC and TbBDC.

Sample name	S_{BET} (m^2/g)	Total pore volume (cm^3/g)	Average pore diameter
EuBDC	14.75	0.111	4.9
TbBDC	14.60	0.099	2.8

Table S4. pH values measured in the suspensions.

**Ions**	**pH**
K (I)	5.93
Na (I)	5.98
NH_4 (I)	5.84
Ca (II)	5.95
Mg (II)	6.31
Zn (II)	5.78

Cu (II)	4.48
Ba (II)	5.95
Ni (II)	4.05
Mn (II)	4.21
Pb (II)	5.53
Cd (II)	3.97
As (III)	6.39
Cr (III)	3.99
Fe (III)	3.69
Ag (I)	4.02
Cl^-	5.93
NO_3^-	5.98
SO_4^{2-}	5.84
SCN^-	6.30
F^-	6.14
$C_2O_4^{2-}$	6.44
MnO_{4-}	5.90
$CrO_4^{2-}/Cr_2O_7^{2-}$	5.10
Blank	5.86

Table S5. Summary of Stern–Volmer data.

TbBDC	Ksv linear, M^{-1}	Ksv exp, M^{-1}	Kd,M^{-1}	Ks, M^{-1}	LOD, mM
Ag(I)	8322	19.61			0.0881
Fe(III)	8924	6903	881	4777	0.0325
$Cr_2O_7^{2-}/CrO_4^{2-}$	2749	2093			0.0495
EuBDC	Ksv linear, M^{-1}	Ksv exp, M^{-1}	Kd,M^{-1}	Ks, M^{-1}	LOD, mM
Ag(I)	2911	6.908			0.1043
Cr(III)	3440	2.76			0.2130

LOD	3σ/slope
σ	STDV
slope	linear dependancy of the intensity to concentration
